# Predicting Serum Levels of Lithium-Treated Patients: A Supervised Machine Learning Approach

**DOI:** 10.3390/biomedicines9111558

**Published:** 2021-10-28

**Authors:** Chih-Wei Hsu, Shang-Ying Tsai, Liang-Jen Wang, Chih-Sung Liang, Andre F. Carvalho, Marco Solmi, Eduard Vieta, Pao-Yen Lin, Chien-An Hu, Hung-Yu Kao

**Affiliations:** 1 Department of Psychiatry, Kaohsiung Chang Gung Memorial Hospital and Chang Gung University College of Medicine, Kaohsiung 83301, Taiwan; paoyenlin@gmail.com (P.-Y.L.); adventurer1163@gmail.com (C.-A.H.); 2 Department of Computer Science and Information Engineering, National Cheng Kung University, Tainan 70101, Taiwan; 3 Department of Psychiatry, School of Medicine, College of Medicine, Taipei Medical University, Taipei 110301, Taiwan; tmcpsyts@tmu.edu.tw; 4 Department of Psychiatry and Psychiatric Research Center, Taipei Medical University Hospital, Taipei Medical University, Taipei 110301, Taiwan; 5 Department of Child and Adolescent Psychiatry, Kaohsiung Chang Gung Memorial Hospital, Chang Gung University College of Medicine, Kaohsiung 83301, Taiwan; wangliangjen@gmail.com; 6 National Defense Medical Center, Department of Psychiatry, Beitou Branch, Tri-Service General Hospital, Taipei 112003, Taiwan; lcsyfw@gmail.com; 7 National Defense Medical Center, Department of Psychiatry, Taipei 114201, Taiwan; 8 IMPACT (Innovation in Mental and Physical Health and Clinical Treatment) Strategic Research Centre, School of Medicine, Barwon Health, Deakin University, Geelong, VIC 3216, Australia; andrefc7@hotmail.com; 9 Psychiatry Department, University of Ottawa, Ottawa, ON K1N 6N5, Canada; marco.solmi83@gmail.com; 10 The Ottawa Hospital, University of Ottawa, Ottawa, ON K1H 8L6, Canada; 11 Clinical Epidemiology Program, Ottawa Hospital Research Institute (OHRI), University of Ottawa, Ottawa, ON K1N 6N5, Canada; 12 Bipolar and Depressive Disorders Unit, Hospital Clinic, IDIBAPS, CIBERSAM, University of Barcelona, 08036 Barcelona, Catalonia, Spain; EVIETA@clinic.cat; 13 Institute for Translational Research in Biomedical Sciences, Kaohsiung Chang Gung Memorial Hospital, Kaohsiung 83301, Taiwan

**Keywords:** bipolar disorder, lithium, machine learning, random forest, support vector machine, therapeutic drug monitoring, XGBoost

## Abstract

Routine monitoring of lithium levels is common clinical practice. This is because the lithium prediction strategies available developed by previous studies are still limited due to insufficient prediction performance. Thus, we used machine learning approaches to predict lithium concentration in a large real-world dataset. Real-world data from multicenter electronic medical records were used in different machine learning algorithms to predict: (1) whether the serum level was 0.6–1.2 mmol/L or 0.0–0.6 mmol/L (binary prediction), and (2) its concentration value (continuous prediction). We developed models from 1505 samples through 5-fold cross-validation and used 204 independent samples to test their performance by evaluating their accuracy. Moreover, we ranked the most important clinical features in different models and reconstructed three reduced models with fewer clinical features. For binary and continuous predictions, the average accuracy of these models was 0.70–0.73 and 0.68–0.75, respectively. Seven features were listed as important features related to serum lithium levels of 0.6–1.2 mmol/L or higher lithium concentration, namely older age, lower systolic blood pressure, higher daily and last doses of lithium prescription, concomitant psychotropic drugs with valproic acid and -pine drugs, and comorbid substance-related disorders. After reducing the features in the three new predictive models, the binary or continuous models still had an average accuracy of 0.67–0.74. Machine learning processes complex clinical data and provides a potential tool for predicting lithium concentration. This may help in clinical decision-making and reduce the frequency of serum level monitoring.

## 1. Introduction

Lithium is an important therapeutic drug and is considered an archetypal mood stabilizer for the management of mood disorders or schizoaffective disorder [1,2,3]. Many international guidelines recommend lithium as a first-line drug, especially for bipolar disorder [4,5]. Even though lithium has clear beneficial effects, the therapeutic range of its serum levels is narrow (<1.2 mmol/L) [6]. Due to the narrow therapeutic index of lithium, a routine monitoring of its serum levels is suggested. However, the need for frequent monitoring may limit the clinical prescription of lithium. For example, in the acute phase of treatment, clinicians first prescribed an initial low-dose lithium dosage regimen that was titrated upwards based on the serum level and clinical response. This approach usually takes days to weeks to reach the optimal serum concentration, which may delay the therapeutic response [7]. Moreover, in the maintenance phase of lithium therapy, the need for frequent blood draws to obtain serum levels may reduce patients’ willingness to use lithium [3,8,9].

In the past few decades, some studies have attempted to solve this issue and provided formulas to calculate the expected steady-state lithium levels for a specific dose [10]. However, these studies have great limitations that hinder their clinical application in real-world settings. For example, the sample size of these previous studies is often small (usually <200 participants) [11,12,13]; while others have included patients in a conditional experimental environment, rather than patients from a real-world clinical environment [10]; prediction errors of these models were not enough to meet clinical needs (i.e., root-mean-square error (RMSE) ≥0.37 mmol/L) [14]. Recently, algorithm-driven machine learning models have been developed as important tools in mental health field [15,16,17]. These algorithms may provide programs that optimize performance under the guidance of training experience, for example, using gender-specific gene expression biomarkers to predict lithium treatment response [18]. Furthermore, with the increasing popularity of big data, such as data from electronic medical records (EMRs), large-scale datasets provide machine learning for training and have the potential to make better prediction models of serum lithium levels more likely [19]. After independent replication, these models may become clinically useful in routine psychiatric care.

This study used a large-scale blood sample derived from EMRs and different algorithmic machine learning methods to develop predictive models of patients’ serum lithium levels. This study aimed to predict serum lithium levels at a therapeutic level of 0.6–1.2 mmol/L (binary prediction) and blood concentration value (continuous prediction). For further clinical applications, the importance of the features in these models was ranked, and new reduced models with fewer features were reconstructed.

## 2. Materials and Methods

### 2.1. Data Source and Study Subjects

**Figure 1:**
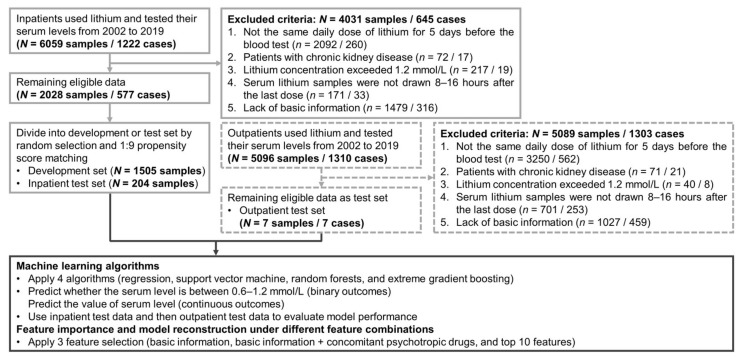
Flowchart of the selection process for this study and outline of analytic procedures.

The research protocol was approved by the institutional review board of Chang Gung Memorial Hospital on 2 February 2021 (No.202100131B0). Figure 1 depicts a flowchart of the selection process and the study design. We used data from the Chang Gung Research Database (CGRD) medical claims between 1 January 2002, and 31 December 2019 to predict serum lithium levels of inpatients with mental disorders. The CGRD is a multicentric EMR, which includes deidentified personal data on demographics (age, sex), medical visits (outpatient and inpatient), pharmacy records (medication type, dosage, frequency, and duration of supply), disease diagnosis by the International Classification of Disease, Ninth Revision (ICD-9) or ICD-10, and laboratory data (hematology tests, biochemical tests, and blood draw time) from seven medical institutes throughout Taiwan [20]. The CGRD covered 14% of inpatients with mental disorders in Taiwan’s total medical population from 1997 to 2010 [21]. Since inpatients routinely take medications under the supervision of nurses (good medication compliance), we only included inpatient pharmacy and laboratory data to develop machine learning algorithms. Patients’ lithium concentration records had to meet the following inclusion criteria: (1) Patients should take the same daily dose for at least 5-day consecutively before the blood test was performed to achieve the steady-state concentration of lithium therapy based on its half-life [22]; (2) Had no chronic kidney disease (i.e., glomerular filtration rate ≥60 mL/min/1.73 m^2^) [23]; (3) Data on serum lithium level ranging from 0.0–1.2 mmol/L [4]; (4) Serum lithium samples were drawn 8–16 h after the last dose [11,24,25]. Additionally, the same set of eligibility criteria to extract additional data on serum lithium samples from outpatients was adopted.

### 2.2. Definition of Outcome Targets, Predictive Features, and Analysis Subject

In this study, two outcome targets for binary and continuous variables were defined. Since serum levels of lithium of 0.6–1.2 mmol/L have been considered the target range for mood disorders [26] and covered most of the recommended treatment guidelines for the manic and maintenance phases of bipolar disorder [4,5,27], binary variable results of the prediction model are divided into 0.6–1.2 mmol/L group (proper treatment) and 0.0–0.6 mmol/L group (undertreatment). Additionally, the serum lithium level is directly regarded as a target for continuous outcomes.

Based on the results of previous studies on lithium interaction and the available data in the database [10,28], we included 114 features for analysis, including basic information (age, sex, height, weight, blood pressure, characteristics of lithium prescription), concomitant medications, comorbidities, and laboratory data. The patient’s comorbidity was defined as whether the disease was recorded in the CGRD within 2 years before admission. Concomitant medication was considered if the patient had used other medications within 5-day before the lithium blood test. For different concomitant psychotropic medications, we calculated the ratio of the average daily dose to the defined daily dose (DDD) used for the modeling. The DDD determined by the World Health Organization Collaborating Centers for Drug Statistics Methodology was used to assume the average maintenance dose per day in adults [29]. Laboratory data were extracted at the same time as the lithium blood sample was collected or at other times within a week. Due to the lack of laboratory data for some patients, we kept patients with at least the other characteristics mentioned above. We then adopted k-nearest neighbor (k-NN) imputation to fill in missing values (laboratory data) [30]. We computed the median of the given variable in the five nearest neighbors of a given subject to fill these values. Detailed information on all features and availability rates of laboratory data are provided in Supplementary Tables S1 and S2.

To obtain prediction models with high generalizability, the inpatient data were divided into an internal development (training/validation) set and an external test set (inpatient test set). First, we randomly selected 10% of the entire data as the test set for external validation [15] and then implemented 1:9 propensity score matching (PSM) on the remaining data to obtain the development set [31]. Development set was used to develop the machine learning model and internal validation [15]. Additionally, we were interested in the applicability of machine learning algorithms to outpatients, so the outpatient data were used for the other external test set (outpatient test set). Finally, the development, inpatient test, and outpatient test sets included 1505, 204, and 7 samples, respectively.

### 2.3. Machine Learning Models and Model Evaluation

Five common machine learning algorithms were used in this study (logistic regression (LogR), linear regression (LinR), support vector machine (SVM) with radial basis function kernel, random forests (RF), and extreme gradient boosting (XGBoost) [18,32,33,34,35]. These algorithms were used to develop predictive models of binary outcomes (LogR, SVM, RF, and XGBoost) and continuous outcomes (LinR, SVM, RF, and XGBoost). The LogR model uses probabilities for classification problems with two classes of dichotomous criteria, and the LinR model predicts the continuous value as a weighted sum of the feature inputs. SVM constructs a set of hyperplanes in a higher-dimensional space to achieve the maximum separation distance of the nearest training data points of any class [36]. RF operates by constructing multiple decision trees during training and outputting a comprehensive prediction based on the mean prediction of individual trees (110 trees with a depth of 7 layers were used in the analysis) [37]. XGBoost builds an ensemble of decision trees by iteratively focusing on harder to predict subsets of the training data (200 trees with a depth of five layers were used in the analysis) [38]. We first apply the regularization method to all features to maintain a common scale range (0–1), so that we can avoid distortion of the value range and overfitting and improve the prediction accuracy of the model. Then, we used the 5-fold cross-validation method to develop the above machine learning models and evaluate their performance through two test sets.

Different performance parameters were calculated for binary and continuous outcomes. We mainly used sensitivity, specificity, the area under the curve of receiver operator characteristic (AUC-ROC), and accuracy for binary prediction and mean absolute error (MAE), mean square error (MSE), RMSE, and accuracy for continuous prediction. For better clinical practice, we defined the accuracy of continuous results (i.e., if the difference between the predicted value and true value is within 0.2 mmol/L, the predicted result will be regarded as a correct prediction). See Supplementary Tables S4 and S5 for all performance parameters. In addition, we conducted Y randomization (Y scrambling) test on the continuous result prediction model to ensure its robustness [39,40]. The lithium concentration value was randomly shuffled once, and a new prediction model was developed using the original features. If the primary predictive model is acceptable, the new predictive model is expected to have a lower R^2^ value than the primary model.

### 2.4. Model Interpretation and Statistical Analysis

We used three methods to rank the importance of features and interpret the model predictions. First, LogR and LinR adopt the least absolute shrinkage and selection operator (LASSO) algorithm. LASSO is a regression analysis that uses the L1 constraint to perform variable selection [41]. Second, we used a backward stepwise method to determine the importance order among the features. This process uses a series of steps to allow features to leave the SVM model one at a time, which allows for interactions between residual features [42]. Third, RF and XGBoost chose Shapley additive explanation (SHAP) to interpret the model predictions. SHAP comes from game theory, and its interpretation is based on the SHAP value of each feature, which represents the contribution of the feature to predicting the event risk. A positive or negative SHAP value indicates that the corresponding feature has an increase or decrease in the lithium concentration, respectively. The SHAP value of each variable is additive, allowing the contribution of each variable to be converted into a part of the output classification probability [43].

We selected three different feature combinations to reconstruct the prediction model to evaluate the clinical applicability of the model with fewer variables. First, we only applied basic information in prediction Model 1. Second, we combined basic information and concomitant psychotropic medications as predictive features in Model 2. Finally, we used the top 10 ensemble features (close to 10% of 114 features) in Model 3, selected from the feature importance ranking results of the different machine learning algorithms mentioned above (consensus features obtained by combining the results of different models).

The independent t-test and χ^2^ test were used to compare the baseline characteristics of the development and test data and the difference between the primary model and the secondary analysis in different feature combinations. We also used a one-way analysis of variance to check the accuracy differences between the four prediction models. All statistical analyses were performed using SAS software (v. 9.4; SAS Institute Inc., Cary, NC, USA). Statistical significance was set at *p*-value < 0.05. All machine learning models were established with Windows Python 3.8 (scikit-learn package v. 0.24.2), and the codes were provided on the GitHub website (github.com/harwic/LithML01) accessed on 18 October 2021.

## 3. Results

### 3.1. Characteristics of Study Participants

A total of 1709 inpatient data were included in this study (mean age, 43.1 years, 45.8% female). Table 1 presents further clinical characteristics after PSM. There was no significant difference between development and inpatient test data. The demographic data of the seven outpatient data are listed in Supplementary Table S3.

### 3.2. Predictive Model Performance

Table 2 first shows the binary outcomes (0.6–1.2 mmol/L vs. 0.0–0.6 mmol/L) of the four algorithms. For inpatient test data, these models had high sensitivity (LogR, 0.89; SVM, 0.94; RF, 0.96; XGBoost, 0.90), low specificity (LogR, 0.43; SVM, 0.32; RF, 0.22; XGBoost, 0.38), with an average AUC-ROC exceeding 0.75 (LogR, 0.75; SVM, 0.76; RF, 0.78; XGBoost, 0.78). The average accuracy of the four algorithms was 0.70–0.73 with no significant differences between them (F = 2.36, *p* = 0.110, data not shown). When the four models were applied to outpatient test data, their model performances were similar to the inpatient test data, such as average accuracy (inpatient: 0.70–0.73; outpatient: 0.77–1.00). The detailed model performance of the binary results is listed in Supplementary Table S4. Table 2 shows the continuous results of the four algorithms. For the inpatient test data, the average MAE, MSE, and RMSE of the four algorithms was 0.14–0.16 mmol/L, 0.03–0.04 mmol/L, and 0.17–0.20 mmol/L, respectively. The average accuracy was 0.68–0.75, and the SVM had the highest accuracy (F = 15.52, *p* < 0.001, data not shown). When the four models were used to predict outpatient test data, the average accuracy was 0.67–0.78, and no differences were observed between the four models (F = 1.33, *p* = 0.299, data not shown). Supplementary Table S5 shows the detailed model performance of continuous results. Additionally, the R^2^ of the new models had lower values than the primary models after Y randomization test (new models: 0.117–0.148, primary models: 0.209–0.370, data not shown).

### 3.3. Feature Importance and Model Performance under Different Feature Combinations

Table 3 summarizes the top 10 features of the different algorithms. Among the top 10 ensemble features of the binary and continuous algorithm models, seven features appeared together and were associated with higher lithium concentrations: older age, lower systolic blood pressure, higher daily and last doses of lithium prescription, concomitant psychotropic medications with valproic acid and -pines drugs, and comorbid substance-related disorders. Of these seven features, the daily dose of lithium prescription and age were the first two important features in predicting serum lithium levels. For detailed information on feature selection, such as LASSO, backward stepwise, and SHAP, as well as the importance ranking and positive-negative relationship of all features of different algorithm models, see Supplementary Materials.

**Figure 2:**
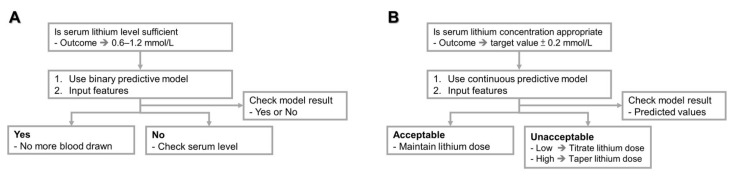
The suggested workflow for clinicians to use the predictive models of this study. (**A**) binary prediction, (**B**) continuous prediction.

Supplementary Figure S9A shows the accuracy of binary outcomes using four algorithms with different feature combinations. The accuracy could be above 0.70 (range from 0.70 to 0.73) regardless of the algorithm or feature combination used. The predictive performance of the three new models was similar to that of the primary model, and no statistical differences were observed. Moreover, Supplementary Figure S9B shows the accuracy of continuous outcomes (0.67–0.75). For the LinR and SVM algorithms, the accuracy of the primary model using all features was significantly higher than that of Model 1/Model 2 and Model 1/Model 3, respectively. However, the models with fewer features performed nearly as well as the primary model in the RF and XGBoost algorithms. Additionally, detailed performance data for various feature combinations are shown in Supplementary Materials. In addition, Figure 2 summarizes the step-by-step decisions recommended for clinicians using the binary or continuous predictive model of this study.

## 4. Discussion

This study used machine-learning algorithm-driven models to predict serum lithium levels. We collected a large number of blood samples from hospitalized patients to develop machine learning models and used independent inpatient and outpatient data to make predictions. Whether it is binary prediction or continuous prediction, the accuracy was 67–100% (binary, 70–100%; continuous: 67–78%). Moreover, we analyzed the feature rankings of these models and found seven important features. Furthermore, we reduced the number of features required to reconstruct the prediction model. The accuracy of most of the new models is close to that of the primary model, with no significant difference.

Several studies have developed predictive lithium dose equations [11,12,13,25,44,45]. Compared to previous studies, this study with large-scale blood sample data in real-world settings is more complicated. For example, participants in this study took various concomitant drugs, such as mood stabilizers, antidepressants, and antipsychotics, which may interact with the pharmacokinetics of lithium (Table 1) [28]. Under such an arduous task, this study has better sensitivity (previous studies, 0.80–0.90; inpatient data result, 0.89–0.96) [14] or RMSE (previous studies, 0.21–0.59 mmol/L; inpatient data result, 0.17–0.20 mmol/L) [14,46], but lower specificity (previous studies, 0.67–0.76; inpatient data result, 0.22–0.43) [14], or higher MAE (previous studies, 0.13 mmol/L; inpatient data result, 0.14–0.16 mmol/L) [25]. Furthermore, these primary models of the inpatient data performed equal or better in most of the outpatient data (Supplementary Tables S4 and S5), indicating that our model has a certain generalization. Notably, the SVM algorithm of the continuous model exhibited the best performance in the inpatient test data among the four algorithms. Clinicians may consider using the SVM algorithm first to predict an inpatient’s lithium concentration.

Compared with previous studies, the predictive performance of the current machine learning algorithm-driven model should be acceptable and useful for real-world clinical practice (Figure 2). Here, we assume that two clinical scenarios are recommended for these predictive tools. First, the binary prediction model can help clinicians track whether lithium treatment reaches the therapeutic concentration in the outpatient setting and reduces the frequency of blood draw. Many studies have indicated that patients receiving lithium treatment should monitor their plasma concentration regularly throughout their life [47,48], but this is a burden on patients. The proportion of patients who receive regular blood lithium monitoring is often lower than the recommended standard [8,9,49]. Therefore, we can use patient information to predict whether the level of lithium treatment is sufficient. If the prediction is sufficient, we may not draw blood (higher sensitivity); otherwise, we draw blood to recheck the lithium concentration (lower specificity). Second, the continuous predictive model can help clinicians adjust the medications of their patients. The current model of this study can estimate the concentration value and increase or decrease the daily dose of lithium to reach the appropriate therapeutic range [6].

The interpretable machine learning model is an important issue; hence, this study summarizes the top 10 features of different algorithms with binary and continuous outcomes. First, it is easy to understand that increasing daily and last doses of lithium elevate its serum level. Second, our study found that the older the age, the higher the lithium concentration. There is evidence that older people have lower lithium clearance, which may explain the results of this study [50,51]. Third, the simultaneous use of valproic acid or -pines drugs is also an important feature in predicting serum lithium levels. Previous clinical trials have shown that valproic acid or quetiapine may interact with lithium and slightly increase serum concentration [28,52,53]. Some studies also indicated that lithium plus valproic acid or quetiapine has a better therapeutic response than lithium monotherapy [54,55]. Compared to lithium monotherapy, lithium plus valproic acid or -pine drugs can enhance therapeutic effects by increasing lithium concentration, partially supporting our findings. In addition, some case reports have found that the combined use of lithium and antipsychotics can cause neurotoxicity, but serum lithium levels may not increase at the same time [56,57,58]. The feature importance ranking of our model can also partially explain it, that is, different types of antipsychotics have different effects on lithium concentration. For example, the most influential drugs in our models are -pine drugs (Supplementary Tables S6 and S7). Fourth, a higher systolic blood pressure reduces the lithium concentration. Increased blood pressure is related to the excessive activity of sodium-lithium countertransport in red blood cells, leading to a decrease in serum lithium levels [59,60]. Finally, a previous study reported that drinking alcohol increases lithium concentration [61], which may reflect the importance of substance use disorders in predictive models. In summary, factors affecting lithium concentration proved by previous studies may play an important predictive role in the machine learning model of real-world data. Furthermore, it is worth noting that true indicators of kidney function, such as blood urea nitrogen or serum creatinine, are not a priority feature to predict lithium concentration in this study [10]. This may be due to the inclusion criteria (glomerular filtration rate ≥60 mL/min/1.73 m^2^), indicating that the renal function of the study participants was relatively normal.

For further clinical applications, we attempted to reduce the features of the algorithm. Even though the new model and the primary model had differences in the accuracy performance of the different algorithms, the overall accuracy of all new models was approximately 70%. This finding inspired us to consider using basic information with or without concomitant psychotropic drugs to predict serum lithium levels of the patient. Furthermore, using only the top 10 features in the predictive model may also be another option. Our findings show that this flexible feature combination of algorithm-driven machine learning models is clinically more practical. For example, clinicians can only use 10 predictors, such as basic information or the top 10 features mentioned in this study, to build a simple model with predictive performance similar to the original model that used 114 predictors.

This study had several limitations. First, we used PSM to select independent inpatient data to reduce the bias of background characteristics between development and test data [16], but collecting development and test data from the same dataset may reduce the generalizability of our models. Second, we extracted outpatient data as another test set to verify reproducibility [16]. Compared to the inpatient test set, the model performance was still acceptable; however, the outpatient test set only included seven blood samples, which may reduce validity. Third, we extracted the patient’s laboratory data with a 1-week buffer period and used the k-NN method to fill in the missing values. What we do may obscure the true value of individual laboratory data and reduce its usefulness in algorithms. For example, renal function is not a priority feature in the current model compared to a previous study [10]. Finally, this study excluded some extreme or outlier data from the database, such as patients with chronic kidney disease, serum lithium levels exceeding 1.2 mmol/L, or blood samples collected less than 8 h or more than 16 h after the last dose; the domain of applicability of our models cannot be extended to those with these conditions [39,62].

## 5. Conclusions

We used real-world EMR data to develop machine learning models to predict serum lithium levels. The average accuracy of binary results or continuous results was 68–75%. Older age, lower systolic blood pressure, higher daily and last doses of lithium prescription, concomitant psychotropic medications with valproic acid and -pines drugs, and comorbid substance-related disorders were important features associated with higher lithium concentrations. We altered the prediction models with fewer features, and the average accuracy was still close to 70%. Our model processed more complex clinical data and provided useful clinical tools for predicting serum lithium levels.

## Figures and Tables

**Table 1:** Characteristics of lithium-treated patients, comparing inpatient development data and inpatient test data.

Characteristics	Development (*n* = 1505)	Test (*n* = 204)	*t* or χ²	*p*
Lithium serum levels, mmol/L	0.69 ± 0.21	0.70 ± 0.22	−0.51	0.612
**Basic Information**				
Age, year	43.13 ± 13.70	42.95 ± 13.82	0.18	0.856
Sex, female	696 (46.25)	87 (42.65)	0.94	0.333
**Clinical Characteristics**				
Height, m	1.64 ± 0.08	1.64 ± 0.09	−0.90	0.370
Weight, kg	69.64 ± 14.73	70.35 ± 14.54	−0.65	0.517
Systolic blood pressure, mmHg	122.00 ± 10.86	122.30 ± 10.42	−0.38	0.706
Diastolic blood pressure, mmHg	76.28 ± 7.48	76.21 ± 7.76	0.14	0.890
**Lithium Prescription**				
Daily dose, mg/day	867.70 ± 266.70	896.30 ± 257.20	−1.44	0.148
Dosing frequency, time/day	2.57 ± 0.78	2.60 ± 0.73	−0.57	0.568
Last dose, mg	354.60 ± 125.20	362.50 ± 131.30	−0.84	0.401
Time interval, hour	13.08 ± 1.44	13.11 ± 1.32	−0.26	0.796
**Concomitant Psychotropic Drugs**				
**Mood Stabilizers**				
Carbamazepine	73 (4.85)	11 (5.39)	0.11	0.737
Lamotrigine	64 (4.25)	7 (3.43)	0.30	0.581
Topiramate	110 (7.31)	15 (7.35)	0.001	0.982
Valproic acid	524 (34.82)	67 (32.84)	0.31	0.578
**Antidepressants**				
SSRI	175 (11.63)	24 (11.76)	0.003	0.954
SNRI	88 (5.85)	10 (4.90)	0.30	0.586
Trazodone	38 (2.52)	4 (1.96)	0.24	0.625
Mirtazapine	45 (2.99)	7 (3.43)	0.12	0.731
Bupropion	62 (4.12)	8 (3.92)	0.02	0.894
Agomelatine	48 (3.19)	7 (3.43)	0.03	0.854
**Antipsychotics**				
Typical antipsychotics	177 (11.76)	23 (11.27)	0.04	0.839
The benzamides	49 (3.26)	7 (3.43)	0.02	0.895
The -dones	295 (19.60)	49 (24.02)	2.18	0.140
The -pines	1033 (68.64)	129 (63.24)	2.41	0.121
Aripiprazole	157 (10.43)	23 (11.27)	0.14	0.713
**Anxiolytics, Sedatives, or Hypnotics**				
Benzodiazepines	1252 (83.19)	169 (82.84)	0.02	0.901
Non-benzodiazepines	208 (13.82)	32 (15.69)	0.52	0.472
Acetylcholinesterase inhibitors	12 (0.80)	1 (0.49)	0.22	0.636
**Mental Disorders**				
Bipolar disorders	1108 (73.62)	150 (73.53)	0.001	0.978
**Laboratory Data**				
Serum creatinine, mg/dL	0.79 ± 0.16	0.80 ± 0.16	0.12	0.469
BUN, mg/dL	9.99 ± 2.64	9.97 ± 2.58	−0.72	0.902

Abbreviations: BUN; blood urea nitrogen; SNRI, serotonin norepinephrine reuptake inhibitor; SSRI, selective serotonin reuptake inhibitor. Data was expressed as N (percentage) or mean ±standard deviation. Time interval is the time between the blood draw and last lithium dose.

**Table 2:** The model performance of the binary and continuous outcomes between different algorithms in inpatient test data.

Binary	LogR	SVM	RF	XGBoost
Sensitivity	0.89 (0.84–0.93)	0.94 (0.91–0.97)	0.96 (0.95–0.97)	0.90 (0.87–0.94)
Specificity	0.43 (0.36–0.51)	0.32 (0.24–0.41)	0.22 (0.13–0.31)	0.38 (0.34–0.41)
AUC-ROC	0.75 (0.73–0.76)	0.76 (0.74–0.77)	0.78 (0.75–0.81)	0.78 (0.74–0.81)
Accuracy	0.73 (0.71–0.75)	0.73 (0.71–0.75)	0.70 (0.68–0.73)	0.72 (0.70–0.74)
**Continuous**	**LinR**	**SVM**	**RF**	**XGBoost**
MAE	0.16 (0.16–0.16)	0.14 (0.13–0.15)	0.15 (0.15–0.16)	0.15 (0.15–0.16)
MSE	0.04 (0.04–0.04)	0.03 (0.03–0.03)	0.04 (0.03–0.04)	0.04 (0.04–0.04)
RMSE	0.20 (0.19–0.20)	0.17 (0.17–0.18)	0.19 (0.18–0.19)	0.19 (0.19–0.20)
Accuracy	0.69 (0.68–0.70)	0.75 (0.71–0.79)	0.68 (0.67–0.70)	0.68 (0.67–0.70)

Abbreviations: AUC-ROC, area under the curve of receiver operator characteristic; LinR, linear regression; LogR, logistic regression; MAE, mean absolute error; MSE, mean-square error; RF, random forests; RMSE, root-mean-square error; SVM, support vector machine; XGBoost, extreme gradient boosting.

**Table 3:** Top 10 features of the binary and continuous outcomes between different algorithms.

Binary	Ensemble	LogR	SVM	RF	XGBoost
Top 1	Daily dose *	Daily dose	Daily dose	Daily dose	Daily dose
Top 2	Age *	MCHC	Topiramate	MCHC	Age
Top 3	Last dose *	Valproic acid	NSAIDs	Last dose	Valproic acid
Top 4	The -pines *	Renal diseases	Hyperlipidemia	Dosing frequency	Height
Top 5	Valproic acid *	Age	Elimination disorders	Age	Time interval
Top 6	Weight	Weight	Age	The -pines	Benzodiazepines
Top 7	SBP *	Substance use disorders	The -pines	Valproic acid	RBC
Top 8	Hypertension	Hypertension	Time interval	Benzodiazepines	WBC
Top 9	MCHC	The -pines	Sleep-wake disorders	Hemoglobin	MCHC
Top 10	Substance use disorders *	Last dose	Last dose	Serum creatinine	RDW-SD
**Continuous**	**Ensemble**	**LinR**	**SVM**	**RF**	**XGBoost**
Top 1	Daily dose *	Daily dose	Daily dose	Daily dose	Daily dose
Top 2	Age *	Valproic acid	Age	Age	Age
Top 3	Valproic acid *	Age	Beta blockers	Valproic acid	Valproic acid
Top 4	The -pines *	Weight	Hyperlipidemia	Weight	The -pines
Top 5	Substance use disorders *	Topiramate	Depressive disorders	RBC	SBP
Top 6	SBP *	ARB	SBP	Height	Weight
Top 7	Beta blockers	MCHC	Valproic acid	RDW-SD	Height
Top 8	Last dose *	Hypertension	Mild DM	SBP	Time interval
Top 9	Potassium	Ocular bleeding	Sex	Last dose	Topiramate
Top 10	NSAIDs	Substance use disorders	Sleep-wake disorders	Topiramate	Benzodiazepines

Abbreviations: ARB, angiotensin receptor blockers; LinR, linear regression; LogR, logistic regression; MCHC, mean corpuscular hemoglobin concentration; Mild DM = diabetes mellitus without end organ damage; NSAIDs, non-steroidal anti-inflammatory drugs; RBC, red blood cell; RDW-SD, red cell distribution width-standard deviation; RF, random forests; SBP, systolic blood pressure; Substance use disorders = substance-related and addictive disorders; SVM, support vector machine; WBC, white blood cell; XGBoost, extreme gradient boosting. * indicated simultaneous occurrence in the top 10 ensemble features of binary and continuous machine learning models.

## Data Availability

The data that support the findings of this study are not publicly available but can be accessed with permission from the Chang Gung Memorial Hospital in Taiwan.

## Supplementary Materials

The following are available online at https://www.mdpi.com/article/10.3390/biomedicines9111558/s1. Figure S1: LASSO method for selecting the top 10 features in logistic regression, binary outcomes. Figure S2: Backward stepwise method for selecting the top 10 features in support vector machine, binary outcomes. Figure S3: Shapley additive explanations method for selecting the top 10 features in random forests, binary outcomes. Figure S4: Shapley additive explanations method for selecting the top 10 features in extreme gradient boost, binary outcomes. Figure S5: LASSO method for selecting the top 10 features in linear regression, continuous outcomes. Figure S6: Backward stepwise method for selecting the top 10 features in support vector machine, continuous outcomes. Figure S7: Shapley additive explanations method for selecting the top 10 features in random forests, continuous outcomes. Figure S8: Shapley additive explanations method for selecting the top 10 features in extreme gradient boost, continuous outcomes. Table S1: Detailed drug name with codes and diagnostic codes for mental disorders or medical diseases. Table S2: Proportion of laboratory data with no missing test values in inpatient data. Table S3: Characteristics of lithium-treated patients, comparing inpatient test data and outpatient test data. Table S4: Detail information of binary outcomes in inpatient test data and outpatient test data. Table S5: Detail information of continuous outcomes in inpatient test data and outpatient test data. Table S6: 114 feature importance ranking results of the 4 different machine learning algorithms in binary outcomes. Table S7: 114 feature importance ranking results of the 4 different machine learning algorithms in continuous outcomes. Table S8: Detail information of binary outcomes of logistic regression algorithm under different feature combinations. Table S9: Detail information of binary outcomes of support vector machine algorithm under different feature combinations. Table S10: Detail information of binary outcomes of random forests algorithm under different feature combinations. Table S11: Detail information of binary outcomes of extreme gradient boosting algorithm under different feature combinations. Table S12: Detail information of continuous outcomes of linear regression algorithm under different feature combinations. Table S13: Detail information of continuous outcomes of support vector machine algorithm under different feature combinations. Table S14: Detail information of continuous outcomes of random forests algorithm under different feature combinations. Table S15: Detail information of continuous outcomes of extreme gradient boosting algorithm under different feature combinations.
