# Supplementary material for: Predicting Serum Levels of Lithium-Treated Patients: A Supervised Machine Learning Approach
Source: Biomedicines. 2021 Oct 28;9(11):1558. doi: 10.3390/biomedicines9111558 (PMC8615637; doi:10.3390/biomedicines9111558)
Supplement: Supplementary file 1 [file biomedicines-09-01558-s001.zip › biomedicines-1417383-supplementary.pdf]

Supplementary information

Predicting serum levels of lithium-treated patients: A supervised machine learning approach

Running Title: Machine learning to predict lithium concentration

Chih-Wei Hsu, MD[1,2,#], Shang-Ying Tsai, MD, PhD[3,4], Liang-Jen Wang, MD, PhD[5], Chih-Sung Liang, MD[6,7], Andre F. Carvalho, MD, PhD[8], Marco Solmi, MD, PhD[9,10,11], Eduard Vieta, MD, PhD[12], Pao-Yen Lin, MD, PhD[1,13], Chien-An Hu, MS[1], Hung-Yu Kao, PhD[2,#]

[1] Department of Psychiatry, Kaohsiung Chang Gung Memorial Hospital and Chang Gung University College of Medicine, Kaohsiung, Taiwan

[2] Department of Computer Science and Information Engineering, National Cheng Kung University, Tainan, Taiwan

[3] Department of Psychiatry, School of Medicine, College of Medicine, Taipei Medical University, Taipei, Taiwan

[4] Department of Psychiatry and Psychiatric Research Center, Taipei Medical University Hospital, Taipei Medical University, Taipei, Taiwan

[5] Department of Child and Adolescent Psychiatry, Kaohsiung Chang Gung Memorial Hospital and Chang Gung University College of Medicine, Kaohsiung, Taiwan

[6] Department of Psychiatry, Beitou Branch, Tri-Service General Hospital, National Defense Medical Center, Taipei, Taiwan

[7] Department of Psychiatry, National Defense Medical Center, Taipei, Taiwan

[8] IMPACT (Innovation in Mental and Physical Health and Clinical Treatment) Strategic Research Centre, School of Medicine, Barwon Health, Deakin University, Geelong, VIC, Australia

[9] University of Ottawa, Psychiatry Department, Ottawa, Ontario, Canada

[10] The Ottawa Hospital, University of Ottawa, Ottawa, Ontario, Canada

[11] Ottawa Hospital Research Institute (OHRI) Clinical Epidemiology Program University of Ottawa Ottawa Ontario

[12] Bipolar and Depressive Disorders Unit, Hospital Clinic, IDIBAPS, CIBERSAM, University of Barcelona, Barcelona, Catalonia, Spain

[13] Institute for Translational Research in Biomedical Sciences, Kaohsiung Chang Gung Memorial Hospital, Kaohsiung, Taiwan

Content	Page
**Appendix 1 (Supplementary Table S1)**. Detailed drug name with codes and diagnostic codes for mental disorders or medical diseases	3-6
**Appendix 2 (Supplementary Table S2).** Proportion of laboratory data with no missing test values in inpatient data	7
**Appendix 3 (Supplementary Table S3).** Characteristics of lithium-treated patients, comparing inpatient test data and outpatient test data	8
**Appendix 4 (Supplementary Table S4).** Detail information of binary outcomes in inpatient test data and outpatient test data	9
**Appendix 5 (Supplementary Table S5).** Detail information of continuous outcomes in inpatient test data and outpatient test data	10
**Appendix 6 (Supplementary Figure S1).** LASSO method for selecting the top 10 features in logistic regression, binary outcomes	11
**Appendix 7 (Supplementary Figure S2).** Backward stepwise method for selecting the top 10 features in support vector machine, binary outcomes	12
**Appendix 8 (Supplementary Figure S3).** Shapley additive explanations method for selecting the top 10 features in random forests, binary outcomes	13
**Appendix 9 (Supplementary Figure S4).** Shapley additive explanations method for selecting the top 10 features in extreme gradient boost, binary outcomes	14
**Appendix 10 (Supplementary Table S6).** 114 feature importance ranking results of the 4 different machine learning algorithms in binary outcomes	15-18
**Appendix 11 (Supplementary Figure S5).** LASSO method for selecting the top 10 features in linear regression, continuous outcomes	19
**Appendix 12 (Supplementary Figure S6).** Backward stepwise method for selecting the top 10 features in support vector machine, continuous outcomes	20
**Appendix 13 (Supplementary Figure S7).** Shapley additive explanations method for selecting the top 10 features in random forests, continuous outcomes	21
**Appendix 14 (Supplementary Figure S8).** Shapley additive explanations method for selecting the top 10 features in extreme gradient boost, continuous outcomes	22
**Appendix 15 (Supplementary Table S7).** 114 feature importance ranking results of the 4 different machine learning algorithms in continuous outcomes	23-26
**Appendix 16 (Supplementary Table S8).** Detail information of binary outcomes of logistic regression algorithm under different feature combinations	27
**Appendix 17 (Supplementary Table S9).** Detail information of binary outcomes of support vector machine algorithm under different feature combinations	28
**Appendix 18 (Supplementary Table S10).** Detail information of binary outcomes of random forests algorithm under different feature combinations	29
**Appendix 19 (Supplementary Table S11).** Detail information of binary outcomes of extreme gradient boosting algorithm under different feature combinations	30
**Appendix 20 (Supplementary Table S12).** Detail information of continuous outcomes of linear regression algorithm under different feature combinations	31
**Appendix 21 (Supplementary Table S13).** Detail information of continuous outcomes of support vector machine algorithm under different feature combinations	32
**Appendix 22 (Supplementary Table S14).** Detail information of continuous outcomes of random forests algorithm under different feature combinations	33
**Appendix 23 (Supplementary Table S15).** Detail information of continuous outcomes of extreme gradient boosting algorithm under different feature combinations	34
**Appendix 24 (Supplementary Figure S9).** Comparison of accuracy performance between the primary model and different new models under different algorithms.	35

Appendix 1 (Supplementary Table S1). Detailed drug name with codes and diagnostic codes for mental disorders or medical diseases

Drug name	Detail drugs and their Anatomical Therapeutic Chemical (ATC) codes
**Concomitant psychotropic medications**	
**Mood stabilizers**	
Lithium	Lithium (N05AN01)
Carbamazepine	Carbamazepine (N03AF01)
Lamotrigine	Lamotrigine (N03AX09)
Valproic acid	Valproic acid (N03AG01)
Topiramate	Topiramate (N03AX11)
**Antidepressants**	
Selective serotonin reuptake inhibitor	Citalopram (N06AB04), Escitalopram (N06AB10), Fluoxetine (N06AB03), Paroxetine (N06AB05), Sertraline (N06AN06)
Serotonin norepinephrine reuptake inhibitor	Duloxetine (N06AX21), Milnacipran (N06AX17), Venlafaxine (N06AX16)
Trazodone	Trazodone (N06AX05)
Mirtazapine	Mirtazapine (N06AX11)
Bupropion	Bupropion (N06AX12)
Agomelatine	Agomelatine (N06AX22)
**Antipsychotics**	
Typical antipsychotics	Chlorpromazine (N05AA01), Haloperidol (N05AD01), Flupentixol (N05AF01), Fluphenazine (N05AB02), Loxapine (N05AH01), Prochlorperazine (N05AB04), Thioridazine (N05AC02), Zuclopenthixol (N05AF05)
The benzamides	Amisulpride (N05AL05), Sulpiride (N05AL01)
The -dones	Paliperidone (N05AX13), Risperidone (N05AX08), Ziprasidone (N05AE04)
The -pines	Clozapine (N05AH02), Olanzapine (N05AH03), Quetiapine (N05AH04), Zotepine (N05AX11)
Aripiprazole	Aripiprazole (N05AX12)
**Anxiolytics, sedatives, or hypnotics**	
Benzodiazepines	Alprazolam (N05BA12), Chlordiazepoxide (N05BA02), Clobazam (N05BA09), Clonazepam (N05AE01), Diazepam (N05BA01), Estazolam (N05CD04), Fludiazepam (N05BA17), Flurazepam (N05CD01), Lorazepam (N05BA06), Midazolam (N05CD08), Triazolam (N05CD05)
Non-benzodiazepines	Zaleplon (N05CF03), Zolpidem (N05CF02)
**Antidementia**	
Acetylcholinesterase inhibitors	Donepezil (N06DA02), Galantamine (N06DA04), Rivastigmine (N06DA03)
**Concomitant medications (other)**	
**Lipid-lowering agents**	
Statins	Statins (C10AA, C10BA)
Fibrates	Fibrates (C10AB)
**Antidiabetic agents**	
Insulins	Insulins (A10A)
Metformin	Metformin (A10BA02, A10BD)
Sodium-glucose cotransporter 2 inhibitors	Sodium-glucose cotransporter 2 inhibitors (A10BK)
Dipeptidyl peptidase 4 inhibitors	Dipeptidyl peptidase 4 inhibitors (A10BH)
Sulfonylureas	Sulfonylureas (A10BB, A10BD02)
Acarbose	Acarbose (A10BF)

Thiazolidinediones	Thiazolidinediones (A10BG)
Meglitinides	Meglitinides (A10BX)
**Anti-hypertensive medications**	
Angiotensin converting enzyme inhibitors	Angiotensin converting enzyme inhibitors (C09A, C09B)
Angiotensin receptor blockers	Angiotensin receptor blockers (C09C, C09D)
Diuretics	Diuretics (C03)
Beta blockers	Beta blockers (C07)
Calcium channel blockers	Calcium channel blockers (C08)
**Antiplatelet drugs**	
Aspirin	Aspirin (B01AC06)
Others	Others (B01AC07, B01AC22, B01AC30, B01AC91)
**Antiarrhythmic drugs**	
Amiodarone	Amiodarone (C01BD01, C01BD07)
**Non-steroidal anti-inflammatory drugs**	Non-steroidal anti-inflammatory drugs (M01AA, M01AX, M01AB, M01AC, M01AE, M01AG, M01AH)
**Antacids**	Antacids (A02BA, A02BC)

**Mental disorders / Medical diseases**	**The International Classification of Diseases 9th (ICD-9) or 10th (ICD-10)**
**Mental disorders**	
Neurodevelopmental disorders	299, 307.2, 307.3, 307.9, 314, 315, 317, 318, 319, F44.4, F63.3, F70, F71, F72, F73, F78, F79, F80.0, F80.1, F80.2, F80.4, F80.89, F80.9, F81, F82, F84.0, F84.3, F84.5, F84.8, F84.9, F88, F89, F90.1, F90.2, F90.8, F90.9, F95, F98.4, F98.8, H93.25, R41.840, R41.841, R41.843, R41.844, R45.1, R45.81, R45.82, R48.0
Disruptive, impulse-control, and conduct disorders	312.32, 312.33, 312.34, 312.8, 312.9, 313.81, F63.1, F63.2, F63.81, F9
Schizophrenia spectrum and other psychotic disorders	295, 297.0, 297.1, 297.2, 298.3, 298.4, 298.8, 298.9, F20, F22, F23, F25, F28, F2
Bipolar and related disorders	296.0, 296.1, 296.4, 296.5, 296.6, 296.7, 296.80, 296.89, 301.13, F30.1, F30.2, F30.3, F30.4, F30.9, F31, F34
Depressive disorders	296.2, 296.3, 296.99, 300.4, 311, 625.4, F32.0, F32.1, F32.2, F32.3, F32.4, F32.5, F32.9, F33, F34.1, F34.8, F34.9, G43.82, G43.83, N94
Anxiety disorders	300.00, 300.01, 300.02, 300.09, 300.2, 309.21, 313.23, F40, F41, F93.0, F94
Obsessive-compulsive and related disorders	300.3, 300.7, 312.39, 698.4, F42, F45.2, F63.3, F63.89, L98
Somatic symptom and related disorders	300.11, 300.19, 300.7, 300.8, 316, F44.2, F44.4, F44.5, F44.6, F44.7, F45.0, F45.1, F45.2, F45.8, F45.9, F48.8, F54, F68
Elimination disorders	307.6, 307.7, 787.60, 788.30, 788.39, F98.0, F98.1, N39.490, N39.498, R15, R32, R39.8
Sleep-wake disorders	307.4, 327, 347, 780.5, F51, G47.0, G47.1, G47.2, G47.3, G47.4, G47.5, G47.61, G47.69, G47.8, G47
Sexual dysfunctions	302.7, F52.0, F52.1, F52.2, F52.3, F52.4, F52.6, F52.8, F52.9, R3
Substance-related and addictive disorders	291.4, 291.81, 292.0, 292.2, 303, 304, 305, 312.31, F10.10, F10.120, F10.129, F10.20, F10.21, F10.220, F10.229, F10.230, F10.232, F10.239, F10.920, F10.929, F11.10, F11.120, F11.129, F11.20, F11.21, F11.220, F11.229, F11.23, F11.90, F11.920, F11.929, F11.93, F12.10, F12.120, F12.129, F12.20, F12.21, F12.220, F12.229, F12.90, F12.920, F12.929, F13.10, F13.120, F13.129, F13.20, F13.21, F13.220, F13.229, F13.23, F13.90, F13.920, F13.929, F13.93, F14.10, F14.120, F14.129, F14.20, F14.21, F14.220, F14.229, F14.23, F14.90, F14.920, F14.929, F15.10,

	F15.120, F15.129, F15.20, F15.21, F15.220, F15.229, F15.23, F15.90, F15.920, F15.929, F15.93, F16.10, F16.120, F16.129, F16.20, F16.21, F16.220, F16.229, F16.90, F16.920, F16.929, F17.200, F17.201, F17.203, F17.210, F17.211, F17.213, F17.220, F17.221, F17.223, F17.290, F17.291, F17.293, F18.10, F18.120, F18.129, F18.20, F18.21, F18.220, F18.229, F18.90, F18.920, F18.929, F19.10, F19.120, F19.129, F19.20, F19.21, F19.220, F19.229, F19.23, F19.90, F19.920, F19.929, F19.93, F55, F63
Neurocognitive disorders (Except vascular dementia)	F02, F05, G30, G31, G31.0, G31.1, G93.7, 290.1, 290.2, 290.3, 294.1, 294.2, 331.0, 331.1, 331.2, 331.8, 331.9, F03.90, G31.83, G31.85, G31.89, F03.90, G31.83, G31.85, G31.8
Personality disorders	301.0, 301.20, 301.22, 301.4, 301.50, 301.6, 301.7, 301.81, 301.82, 301.83, 301.89, 301.9, F21, F60, F6
Trauma- and stressor-related disorders	308.3, 309.0, 309.24, 309.28, 309.3, 309.4, 309.81, 309.89, 309.9, 313.89, F43.0, F43.1, F43.21, F43.22, F43.23, F43.24, F43.25, F43.8, F43.9, F93.8, F94.1, F94.2, F94.9, F98
Dissociative disorders	300.12, 300.13, 300.14, 300.15, 300.6, F44.0, F44.1, F44.81, F44.9, F48
Feeding and eating disorders	307.1, 307.5, F50, F98.2, F98
Gender dysphoria	302.6, 302.85, F64.1, F64.2, F64.8, F64
Paraphilic disorders	302.2, 302.3, 302.4, 302.81, 302.82, 302.83, 302.84, 302.89, 302.9, F65, F6
**Medical diseases**	
Myocardial infarct	410, 412, I21, I22, I25
Congestive heart failure	398.91, 402.01, 402.11, 402.91, 404.01, 404.03, 404.11, 404.13, 404.91, 404.93, 425.4, 425.5, 425.6, 425.7, 425.8, 425.9, 428, I5
Peripheral vascular diseases	047.1, 093.0, 437.3, 440, 441, 443.1, 443.2, 443.3, 443.4, 443.5, 443.6, 443.7, 443.8, 443.9, 557.1, 557.9, I71, I73.9, I79.0, R02, Z95.8, Z95
Cerebrovascular diseases	362.34, 430, 431, 432, 433, 434, 435, 436, 437, 438, G45.0, G45.1, G45.2, G45.4, G45.8, G45.9, G46, I60, I61, I62, I63, I64, I65, I66, I67.0, I67.1, I67.2, I67.4, I67.5, I67.6, I67.7, I67.8, I67.9, I68.1, I68.2, I68.8, I6
Vascular dementia	290.4, F01
Hemiplegia	334.1, 342, 343, 344.0, 344.1, 344.2, 344.3, 344.4, 344.5, 344.6, 344.9, G04.1, G81, G82.0, G82.1, G82
Chronic pulmonary diseases	416.8, 416.9, 490, 491, 492, 493, 494, 495, 496, 497, 498, 499, 500, 501, 502, 503, 504, 505, 506.4, 508.1, 508.8, J40, J41, J42, J43, J44, J44, J45, J46, J47, J60, J61, J62, J63, J64, J65, J66, J6
Peptic ulcer diseases	531, 532, 533, 534, K25, K26, K27, K28
Liver diseases without sequelae	070.22, 070.23, 070.32, 070.33, 070.44, 070.54, 070.6, 070.9, 570, 571, 573.3, 573.4, 573.8, K70.2, K70.3, K71.7, K73, K74.0, K74.2, K74.3, K74.4, K74.5, K74.6, V42
Liver diseases with sequelae	456.0, 456.1, 456.2, 572.2, 572.3, 572.4, 572.5, 572.6, 572.7, 572.8, K72.1, K72.9, K76.6, K76
Renal diseases	403.01, 403.11, 403.91, 404.02, 404.03, 404.12, 404.13, 404.92, 404.93, 582, 583.0, 583.1, 583.2, 583.3, 583.4, 583.5, 583.6, 583.7, 585, 586, 588.0, N01, N03, N05.2, N05.3, N05.4, N05.5, N05.6, N07.2, N07.3, N07.4, N18, N19, N25, V42.0, V45.1, V5
Connective tissue diseases	446.5, 710.0, 710.1, 710.2, 710.3, 710.4, 725, M05.0, M05.1, M05.2, M05.3, M05.8, M05.9, M06.0, M06.3, M06.9, M32, M33.2, M34, M35
Any cancer	140, 141, 142, 143, 144, 145, 146, 147, 148, 149, 150, 151, 152, 153, 154, 155, 156, 157, 158, 159, 160, 161, 162, 163, 164, 165, 166, 167, 168, 169, 170, 171, 172, 174, 175, 176, 177, 178, 179, 180, 181, 182,

	183, 184, 185, 186, 187, 188, 189, 190, 191, 192, 193, 194, 195, 195.0, 195.1, 195.2, 195.3, 195.4, 195.5, 195.6, 195.7, 195.8, 200, 201, 202, 203, 204, 205, 206, 207, 208, 238.6, C0, C1, C2, C3, C40, C41, C43, C45, C46, C47, C48, C49, C5, C6, C70, C71, C72, C73, C74, C75, C76, C80, C81, C82, C83, C84, C85, C88.3, C88.7, C88.9, C90.0, C90.1, C91, C92, C93, C94.0, C94.1, C94.2, C94.3, C94.51, C94.7, C95, C9
Metastatic solid tumor	196, 197, 198, 199, C77, C78, C79, C8
Acquired immune deficiency syndrome	042, 043, 044, B20, B21, B22, B23, B2
Hypertension	401, 402, 403, 404, 405, I10, I11, I12, I13, I15, N26
Hyperlipidemia	272, E75.2, E75.3, E75.5, E75.6, E77, E78, E88.1, E88.2, E88.8
Diabetes mellitus without end organ damage	250.0, 250.1, 250.2, 250.3, 250.8, 250.9, E10.1, E10.5, E10.9, E11.1, E11.5, E11.9, E13.1, E13.5, E13.9, E14.1, E14.5, E14
Diabetes mellitus with end organ damage	250.4, 250.5, 250.6, 250.7, E10.2, E10.3, E10.4, E11.2, E11.3, E11.4, E13.2, E13.3, E13.4, E14.2, E14.3, E14
Intracranial bleeding	430, 431, 432.0, 432.1, 432.9, 852.0, 852.2, 852.4, 853.0, I60, I61, I62, S06.340A, S06.341A, S06.342A, S06.343A, S06.344A, S06.345A, S06.346A, S06.347A, S06.348A, S06.349A, S06.350A, S06.351A, S06.352A, S06.353A, S06.354A, S06.355A, S06.356A, S06.357A, S06.358A, S06.359A, S06.360A, S06.361A, S06.362A, S06.363A, S06.364A, S06.365A, S06.366A, S06.367A, S06.368A, S06.369A, S06.4x0A, S06.4x1A, S06.4x2A, S06.4x3A, S06.4x4A, S06.4x5A, S06.4x6A, S06.4x7A, S06.4x8A, S06.4x9A, S06.5x0A, S06.5x1A, S06.5x2A, S06.5x3A, S06.5x4A, S06.5x5A, S06.5x6A, S06.5x7A, S06.5x8A, S06.5x9A, S06.6x0A, S06.6x1A, S06.6x2A, S06.6x3A, S06.6x4A, S06.6x5A, S06.6x6A, S06.6x7A, S06.6x8A, S06.6x9
Ocular bleeding	360.43, 362.81, 363.6, 364.4, 372.72, 376.32, 377.42, 379.23, H05.23, H11.3, H21.0, H31.3, H35.6, H43.1, H44.8, H47.0
Intraabdominal bleeding	568.81, 866.01, 866.02, 866.11, 866.12, K66.1, S31.001A, S37.011
Hemopericardium or pericardial bleeding	423.0, I31
Hematoma or compartment syndrome	599.7, M79.A, N30.01, N30.21, N30.31, N30.81, N30.91, R3
Gastrointestinal bleeding	530.7, 531, 531.2, 531.4, 531.6, 532, 533, 534, 535.01, 535.11, 535.21, 535.31, 535.41, 535.51, 535.61, 535.71, 537.83, 537.84, 562.02, 562.03, 562.12, 562.13, 569.3, 569.85, 578, K22.6, K25, K26, K27, K28, K29.01, K29.21, K29.31, K29.41, K29.51, K29.61, K29.71, K29.81, K29.91, K31.811, K31.82, K52.81, K55.21, K56.60, K56.60, K57.01, K57.11, K57.13, K57.21, K57.31, K57.33, K57.81, K57.91, K57.93, K62.5, K92.0, K92.1, K92
Other bleeding	302.7, F52.0, F52.1, F52.2, F52.3, F52.4, F52.6, F52.8, F52.9, R3

Appendix 2 (Supplementary Table S2). Proportion of laboratory data with no missing test values in inpatient data

**Features**	**Inpatient data (N = 2028)**
White blood cell	699 (34.47)
Segment	599 (29.54)
Lymphocyte	599 (29.54)
Monocyte	599 (29.54)
Eosinophil	599 (29.54)
Basophil	599 (29.54)
Red blood cell	614 (30.28)
Hemoglobin	619 (30.52)
Hematocrit	619 (30.52)
Mean corpuscular volume	614 (30.28)
Mean corpuscular hemoglobin	614 (30.28)
Mean corpuscular hemoglobin concentration	614 (30.28)
Red cell distribution width-standard deviation	614 (30.28)
Red cell distribution width-coefficient of variation	468 (23.08)
Platelet	614 (30.28)
Blood urea nitrogen	584 (28.80)
Serum creatinine	638 (31.46)
Aspartate aminotransferase	522 (25.74)
Alanine aminotransferase	481 (23.72)
Natrium	455 (22.44)
Potassium	468 (23.08)

Data was expressed as N (percentage).

Appendix 3 (Supplementary Table S3). Characteristics of lithium-treated patients, comparing inpatient test data and outpatient test data

**Characteristics**	**Inpatient (N = 204)**	**Outpatient (N = 7)**	***t* or χ²**	***p***
**Lithium serum levels**, mmol/l	0.70 ± 0.22	0.59 ± 0.24	1.32	0.188
**Basic information**				
Age, year	42.95 ± 13.82	46.86 ± 6.79	-0.75	0.457
Sex, female	87 (42.65)	3 (42.86)	0.000	0.991
Clinical characteristics				
Height, m	1.64 ± 0.09	1.65 ± 0.07	-0.33	0.745
Weight, kg	70.35 ± 14.54	76.43 ± 20.98	-1.07	0.285
Systolic blood pressure, mmHg	122.30 ± 10.42	130.70 ± 18.74	-2.03	0.043
Diastolic blood pressure, mmHg	76.21 ± 7.76	76.14 ± 10.95	0.02	0.983
Lithium prescription				
Daily dose, mg/day	896.30 ± 257.20	857.10 ± 364.50	0.39	0.696
Dosing frequency, time/day	2.60 ± 0.73	2.43 ± 1.27	0.60	0.547
Last dose, mg	362.50 ± 131.30	385.70 ± 146.40	1.24	0.647
Time interval, hour	13.11 ± 1.32	13.43 ± 1.27	-0.63	0.527
**Concomitant psychotropic drugs**				
Mood stabilizers				
Carbamazepine	11 (5.39)	1 (14.29)	1.00	0.318
Lamotrigine	7 (3.43)	1 (14.29)	2.19	0.139
Topiramate	15 (7.35)	0 (0.00)	0.55	0.457
Valproic acid	67 (32.84)	5 (71.43)	4.48	0.034
Antidepressants				
SSRI	24 (11.76)	0 (0.00)	0.93	0.335
SNRI	10 (4.90)	0 (0.00)	0.36	0.548
Trazodone	4 (1.96)	0 (0.00)	0.14	0.708
Mirtazapine	7 (3.43)	0 (0.00)	0.25	0.618
Bupropion	8 (3.92)	0 (0.00)	0.29	0.593
Agomelatine	7 (3.43)	0 (0.00)	0.25	0.618
Antipsychotics				
Typical antipsychotics	23 (11.27)	0 (0.00)	0.89	0.347
The benzamides	7 (3.43)	0 (0.00)	0.25	0.618
The -dones	49 (24.02)	0 (0.00)	2.19	0.139
The -pines	129 (63.24)	0 (0.00)	11.39	0.001
Aripiprazole	23 (11.27)	0 (0.00)	0.89	0.347
Anxiolytics, sedatives, or hypnotics				
Benzodiazepines	169 (82.84)	0 (0.00)	29.13	<0.0001
Non-benzodiazepines	32 (15.69)	0 (0.00)	1.29	0.255
Acetylcholinesterase inhibitors	1 (0.49)	0 (0.00)	0.03	0.853
**Mental disorders**				
Bipolar disorders	150 (73.53)	5 (71.43)	0.02	0.902
**Laboratory data**				
Serum creatinine, mg/dL	0.80 ± 0.16	0.80 ± 0.12	-0.90	0.970
BUN, mg/dL	9.97 ± 2.58	10.86 ± 2.01	-0.04	0.368

Abbreviations: BUN; blood urea nitrogen; SNRI, serotonin norepinephrine reuptake inhibitor; SSRI, selective serotonin reuptake inhibitor.

Data was expressed as N (percentage) or mean ± standard deviation. Time interval is the time between the blood draw and last lithium dose.

Appendix 4 (Supplementary Table S4). Detail information of binary outcomes in inpatient test data and outpatient test data

Model	TP	TN	FP	FN	TPR (Sensitivity)	TNR (Specificity)	PPV (Precision)	NPV	AUC-ROC	F-score	Accuracy
**LogR**											
Inpatient	118	31	40	15	0.886	0.431	0.745	0.675	0.748	0.809	0.727
(n = 204)	(112-124)	(25-36)	(35-46)	(9-21)	(0.839-0.932)	(0.356-0.506)	(0.726-0.764)	(0.600-0.750)	(0.732-0.764)	(0.792-0.826)	(0.707-0.748)
Outpatient	5	2	0	0	1.000	1.000	1.000	1.000	1.000	1.000	1.000
(n =7)	(5-5)	(2-2)	(0-0)	(0-0)	(1.000-1.000)	(1.000-1.000)	(1.000-1.000)	(1.000-1.000)	(1.000-1.000)	(1.000-1.000)	(1.000-1.000)
**SVM**											
Inpatient	125	23	48	8	0.940	0.324	0.723	0.747	0.759	0.817	0.725
(n = 204)	(121-129)	(17-29)	(42-54)	(4-12)	(0.911-0.969)	(0.241-0.407)	(0.702-0.744)	(0.675-0.819)	(0.744-0.774)	(0.806-0.828)	(0.706-0.745)
Outpatient	5	1	1	0	0.920	0.700	0.887	0.800	0.960	0.902	0.857
(n =7)	(4-5)	(1-2)	(0-1)	(0-1)	(0.784-1.056)	(0.360-1.040)	(0.757-1.016)	(0.460-1.140)	(0.892-1.028)	(0.778-1.026)	(0.680-1.035)
**RF**											
Inpatient	127	16	55	6	0.958	0.220	0.697	0.728	0.784	0.807	0.701
(n = 204)	(126-129)	(10-22)	(49-61)	(4-7)	(0.947-0.969)	(0.134-0.305)	(0.676-0.719)	(0.659-0.797)	(0.754-0.814)	(0.794-0.820)	(0.675-0.727)
Outpatient	5	2	0	0	1.000	0.900	0.967	1.000	1.000	0.982	0.971
(n =7)	(5-5)	(1-2)	(0-1)	(0-0)	(1.000-1.000)	(0.622-1.178)	(0.874-1.059)	(1.000-1.000)	(1.000-1.000)	(0.931-1.032)	(0.892-1.051)
**XGBoost**											
Inpatient	120	27	44	13	0.904	0.377	0.731	0.681	0.775	0.808	0.721
(n = 204)	(116-125)	(24-29)	(42-47)	(8-17)	(0.870-0.938)	(0.344-0.411)	(0.721-0.741)	(0.607-0.756)	(0.741-0.810)	(0.792-0.825)	(0.700-0.741)
Outpatient	4	2	0	1	0.720	0.900	0.950	0.600	0.960	0.811	0.771
(n =7)	(2-5)	(1-2)	(0-1)	(0-3)	(0.498-0.942)	(0.622-1.178)	(0.811-1.089)	(0.286-0.914)	(0.849-1.071)	(0.647-0.975)	(0.569-0.974)

Abbreviations: AUC-ROC, area under the curve of receiver operator characteristic; FN, false negative; FP, false positive; LogR, logistic regression; NPV, negative predictive value; PPV, positive predictive value; RF, random forests; SVM, support vector machine; TN, true negative; TP, true positive; TNR, true negative rate; TPR, true positive rate; XGBoost, extreme gradient boosting.

Appendix 5 (Supplementary Table S5). Detail information of continuous outcomes in inpatient test data and outpatient test data

Model	MAE	MSE	RMSE	R^2	Accuracy
**LinR**					
Inpatient (n = 204)	0.159 (0.156-0.162)	0.038 (0.037-0.040)	0.196 (0.193-0.199)	0.209 (0.187-0.231)	0.690 (0.677-0.703)
Outpatient (n =7)	0.144 (0.136-0.152)	0.031 (0.028-0.034)	0.175 (0.167-0.183)	0.227 (0.153-0.300)	0.667 (0.529-0.805)
**SVM**					
Inpatient (n = 204)	0.140 (0.134-0.145)	0.031 (0.028-0.033)	0.175 (0.167-0.183)	0.370 (0.312-0.427)	0.751 (0.710-0.792)
Outpatient (n =7)	0.151 (0.143-0.159)	0.032 (0.029-0.035)	0.178 (0.170-0.187)	0.198 (0.122-0.274)	0.689 (0.538-0.840)
**RF**					
Inpatient (n = 204)	0.152 (0.147-0.156)	0.035 (0.034-0.037)	0.188 (0.184-0.192)	0.273 (0.245-0.301)	0.680 (0.665-0.696)
Outpatient (n =7)	0.140 (0.129-0.151)	0.027 (0.022-0.033)	0.165 (0.149-0.180)	0.315 (0.181-0.448)	0.756 (0.640-0.871)
**XGBoost**					
Inpatient (n = 204)	0.155 (0.150-0.159)	0.037 (0.036-0.039)	0.193 (0.189-0.196)	0.236 (0.206-0.266)	0.683 (0.671-0.695)
Outpatient (n =7)	0.121 (0.108-0.134)	0.022 (0.018-0.026)	0.149 (0.136-0.163)	0.436 (0.337-0.535)	0.778 (0.680-0.875)

Abbreviations: LinR, linear regression; MAE, mean absolute error; MSE, mean-square error; RF, random forests; RMSE, root-mean-square error; SVM, support vector machine; XGBoost, extreme gradient boosting.

Appendix 6 (Supplementary Figure S1). LASSO method for selecting the top 10 features in logistic regression, binary outcomes

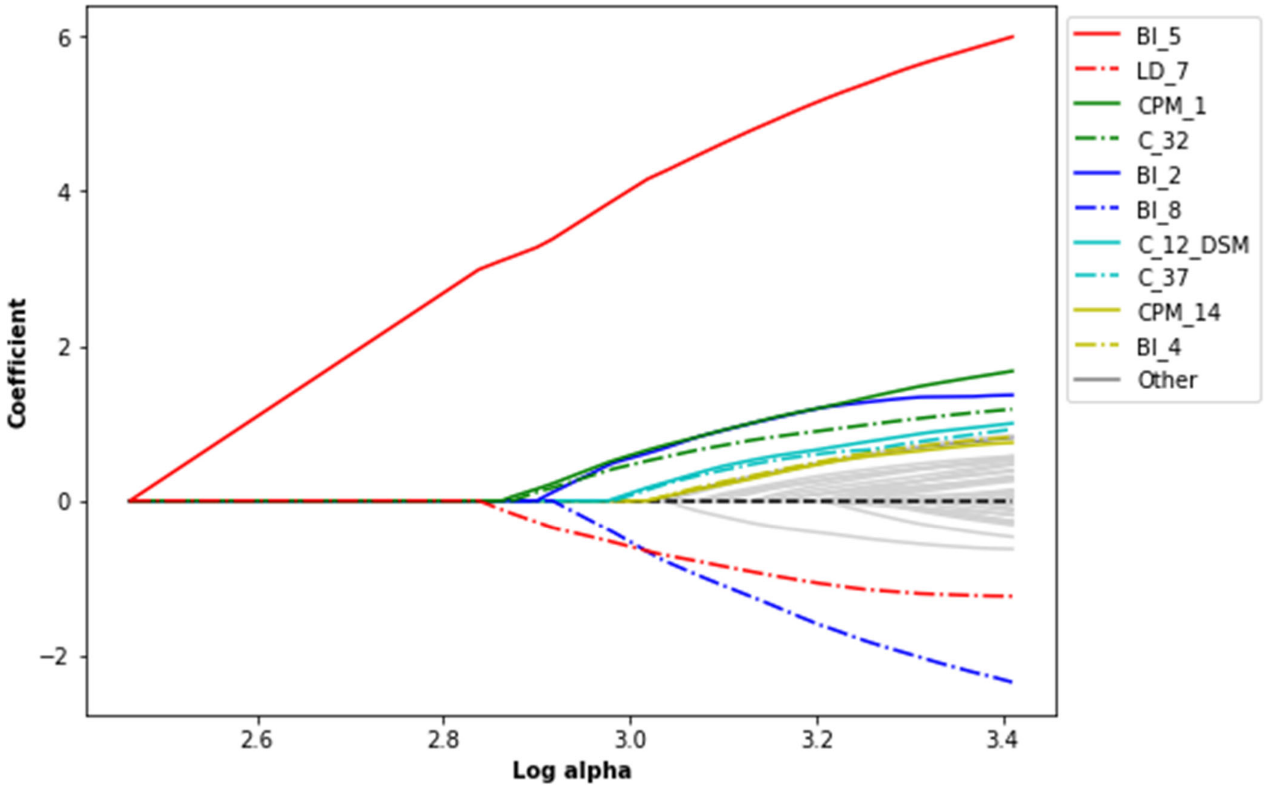

BI_5 = Daily dose, LD_7 = Mean corpuscular hemoglobin concentration, CPM_1 = Valproic acid, C_32 = Renal diseases, BI_2 = Age, BI_8 = Weight, C_12_DSM = Substance-related and addictive disorders, C_37 = Hypertension, CPM_14 = The -pines, BI_4 = Last dose.

Appendix 7 (Supplementary Figure S2). Backward stepwise method for selecting the top 10 features in support vector machine, binary outcomes

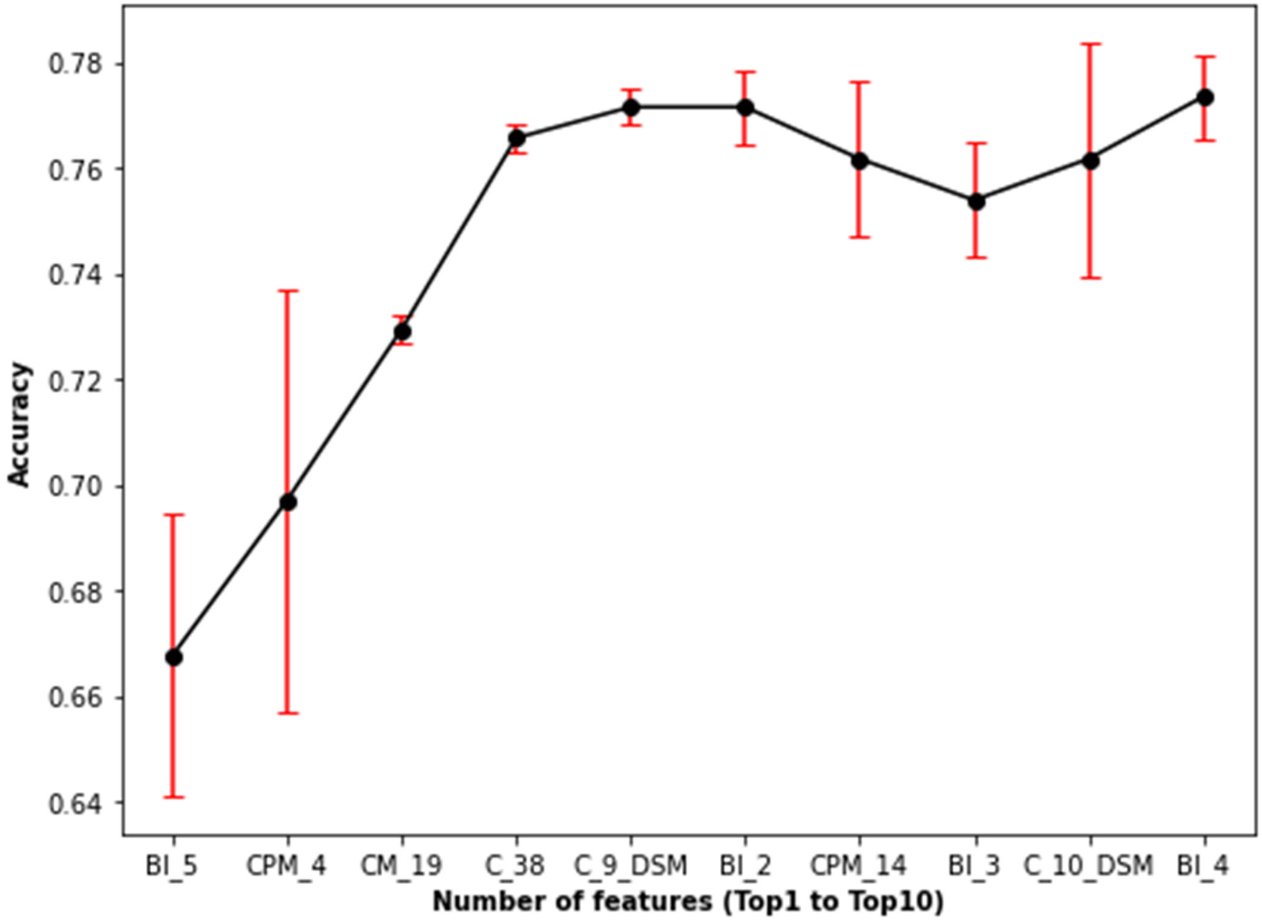

BI_5 = Daily dose, CPM_4 = Topiramate, CM_19 = Non-steroidal anti-inflammatory drugs, C_38 = Hyperlipidemia, C_9_DSM = Elimination disorders, BI_2 = Age, CPM_14 = The -pines, BI_3 = Time interval, C_10_DSM = Sleep-wake disorders, BI_4 = Last dose.

Appendix 8 (Supplementary Figure S3). Shapley additive explanations method for selecting the top 10 features in random forests, binary outcomes

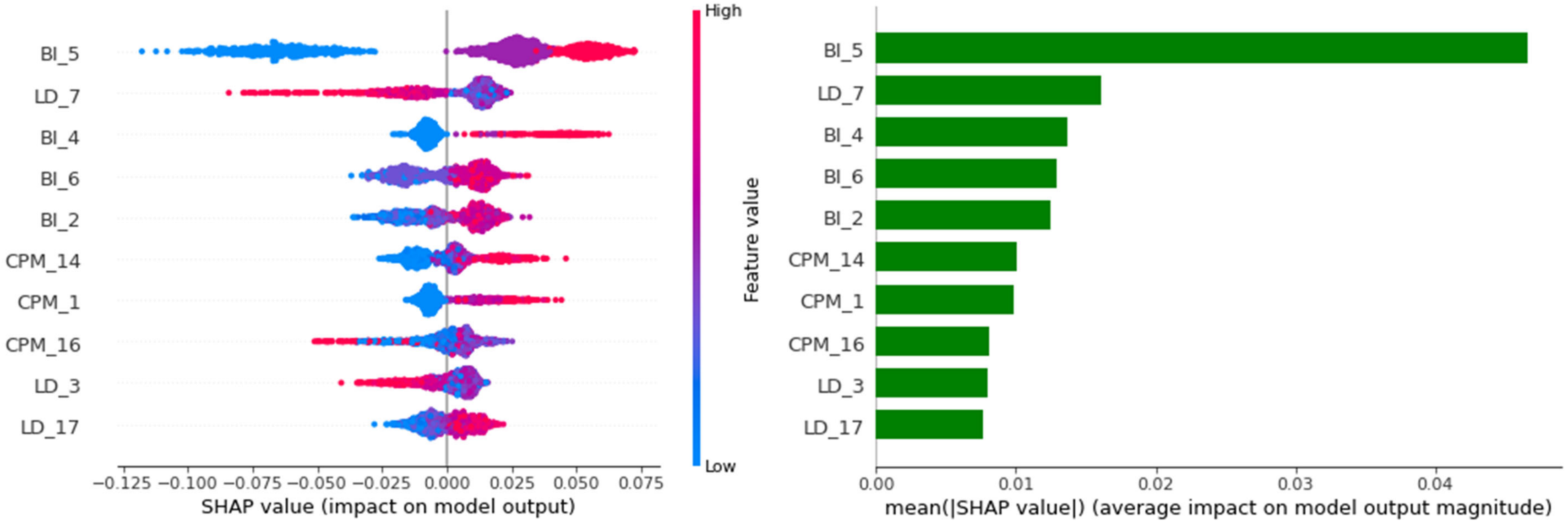

BI_5 = Daily dose, LD_7 = Mean corpuscular hemoglobin concentration, BI_4 = Last dose, BI_6 = Dosing frequency, BI_2 = Age, CPM_14 = The -pines, CPM_1 = Valproic acid, CPM_16 = Benzodiazepines, LD_3 = Hemoglobin, LD_17 = Serum creatinine.

Appendix 9 (Supplementary Figure S4). Shapley additive explanations method for selecting the top 10 features in extreme gradient boost, binary outcomes

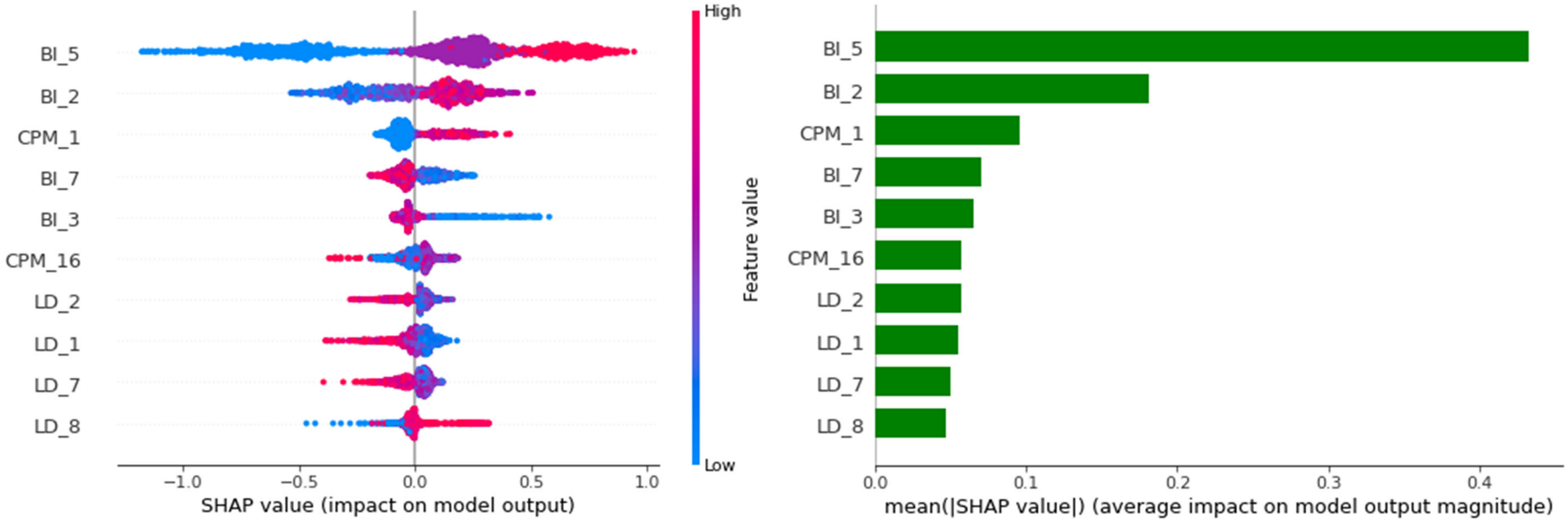

BI_5 = Daily dose, BI_2 = Age, CPM_1 = Valproic acid, BI_7 = Height, BI_3 = Time interval, CPM_16 = Benzodiazepines, LD_2 = Red blood cell, LD_1 = White blood cell, LD_7 = Mean corpuscular hemoglobin concentration, LD_8 = Red cell distribution width-standard deviation.

Appendix 10 (Supplementary Table S6). 114 feature importance ranking results of the 4 different machine learning algorithms in binary outcomes

Rank	Logistic regression	Support vector machine	Random forests	Extreme gradient boosting
1	Daily dose	Daily dose	Daily dose	Daily dose
2	Mean corpuscular hemoglobin concentration (gHb/dL)	Topiramate	Mean corpuscular hemoglobin concentration (gHb/dL)	Age
3	Valproic acid	Non-steroidal anti-inflammatory drugs	Last dose	Valproic acid
4	Renal diseases	Hyperlipidemia	Dosing frequency	Height (m)
5	Age	Elimination disorders	Age	Time interval
6	Weight (Kg)	Age	The -pines	Benzodiazepines
7	Substance-related and addictive disorders	The -pines	Valproic acid	Red blood cell (count/uL)
8	Hypertension	Time interval	Benzodiazepines	White blood cell (count/uL)
9	The -pines	Sleep-wake disorders	Hemoglobin (g/dL)	Mean corpuscular hemoglobin concentration (gHb/dL)
10	Last dose	Last dose	Serum creatinine (mg/dL)	Red cell distribution width-standard deviation(fL)
11	Potassium (mg/dL)	Diabetes mellitus without end organ damage	Red blood cell (count/uL)	Last dose
12	Systolic blood pressure (mmHg)	Hypertension	Weight (Kg)	Weight (Kg)
13	Angiotensin receptor blockers	Bipolar and related disorders	Red cell distribution width-coefficient of variation (%)	The -dones
14	Serotonin norepinephrine reuptake inhibitor	Dosing frequency	White blood cell (count/uL)	Red cell distribution width-coefficient of variation (%)
15	Dipeptidyl peptidase 4 inhibitors	Systolic blood pressure (mmHg)	Height (m)	The -pines
16	Agomelatine	Cerebrovascular diseases	Hematocrit (%)	Topiramate
17	Trauma- and stressor-related disorders	Substance-related and addictive disorders	Natrium (mg/dL)	Serum creatinine (mg/dL)
18	Elimination disorders	Gastrointestinal bleeding	Red cell distribution width-standard deviation(fL)	Natrium (mg/dL)
19	Non-steroidal anti-inflammatory drugs	Valproic acid	Potassium (mg/dL)	Blood urea nitrogen (mg/dL)
20	Selective serotonin reuptake inhibitor	Renal diseases	Systolic blood pressure (mmHg)	Non-steroidal anti-inflammatory drugs
21	Topiramate	Benzodiazepines	Hypertension	Substance-related and addictive disorders
22	Obsessive-compulsive and related disorders	Non-benzodiazepines	The -dones	Alanine aminotransferase (U/L)
23	The -dones	Neurodevelopmental disorders	White blood cell-segment (%)	Hemoglobin (g/dL)
24	Beta blockers	Selective serotonin reuptake inhibitor	Alanine aminotransferase (U/L)	Potassium (mg/dL)
25	Height (m)	Weight (Kg)	Aspartate aminotransferase (U/L)	Dosing frequency
26	Serum creatinine (mg/dL)	White blood cell-monocyte (%)	Blood urea nitrogen (mg/dL)	Systolic blood pressure (mmHg)
27	Trazodone	Personality disorders	Diastolic blood pressure (mmHg)	Platelet (count/uL)
28	Angiotensin converting enzyme inhibitors	Any cancer	White blood cell-monocyte (%)	White blood cell-segment (%)
29	Anxiety disorders	Anxiety disorders	Platelet (count/uL)	Depressive disorders
30	Dissociative disorders	Obsessive-compulsive and related disorders	White blood cell-lymphocyte (%)	Hypertension
31	Hematocrit (%)	Mean corpuscular hemoglobin concentration (gHb/dL)	White blood cell-eosinophil (%)	Aspartate aminotransferase (U/L)
32	Calcium channel blockers	Schizophrenia spectrum and other psychotic disorders	Mean corpuscular volume (fL)	Hyperlipidemia

**33**	Time interval	Beta blockers	Mean corpuscular hemoglobin (pg)	Aripiprazole
**34**	Acquired immune deficiency syndrome	Trauma- and stressor-related disorders	Renal diseases	Diastolic blood pressure (mmHg)
**35**	Ocular bleeding	Feeding and eating disorders	Bipolar and related disorders	Angiotensin receptor blockers
**36**	Disruptive, impulse-control, and conduct disorders	Intracranial bleeding	Substance-related and addictive disorders	White blood cell-eosinophil (%)
**37**	Hyperlipidemia	White blood cell-basophil (%)	Time interval	Beta blockers
**38**	White blood cell-eosinophil (%)	The -dones	White blood cell-basophil (%)	White blood cell-lymphocyte (%)
**39**	Natrium (mg/dL)	Mean corpuscular hemoglobin (pg)	Anxiety disorders	White blood cell-monocyte (%)
**40**	Acetylcholinesterase inhibitors	Angiotensin receptor blockers	Beta blockers	Schizophrenia spectrum and other psychotic disorders
**41**	White blood cell (count/uL)	Hematocrit (%)	Topiramate	Mean corpuscular volume (fL)
**42**	Other bleeding	Depressive disorders	Gastrointestinal bleeding	Gastrointestinal bleeding
**43**	Sodium-glucose cotransporter 2 inhibitors	White blood cell-lymphocyte (%)	Aripiprazole	Hematocrit (%)
**44**	Alanine aminotransferase (U/L)	Serum creatinine (mg/dL)	Trauma- and stressor-related disorders	White blood cell-basophil (%)
**45**	Diabetes mellitus without end organ damage	Sex	Typical antipsychotics	Mean corpuscular hemoglobin (pg)
**46**	Feeding and eating disorders	Metformin	Angiotensin converting enzyme inhibitors	Neurodevelopmental disorders
**47**	Fibrates	Acquired immune deficiency syndrome	Non-steroidal anti-inflammatory drugs	Renal diseases
**48**	Any cancer	Lamotrigine	Depressive disorders	Mirtazapine
**49**	Other antiplatelet	Diuretics	Hyperlipidemia	Non-benzodiazepines
**50**	Aripiprazole	Hemiplegia	Calcium channel blockers	Calcium channel blockers
**51**	Gastrointestinal bleeding	Hemopericardium or pericardial bleeding	Disruptive, impulse-control, and conduct disorders	Antacids
**52**	Hematoma or compartment syndrome	Dissociative disorders	Sex	Carbamazepine
**53**	Hemopericardium or pericardial bleeding	Ocular bleeding	Serotonin norepinephrine reuptake inhibitor	Serotonin norepinephrine reuptake inhibitor
**54**	Intraabdominal bleeding	Chronic pulmonary diseases	Lamotrigine	Feeding and eating disorders
**55**	Intracranial bleeding	Liver diseases with sequelae	Angiotensin receptor blockers	Bupropion
**56**	Metastatic solid tumor	Blood urea nitrogen (mg/dL)	Selective serotonin reuptake inhibitor	Disruptive, impulse-control, and conduct disorders
**57**	Liver diseases with sequelae	White blood cell (count/uL)	Schizophrenia spectrum and other psychotic disorders	Typical antipsychotics
**58**	Hemiplegia	Congestive heart failure	Non-benzodiazepines	Sleep-wake disorders
**59**	Diabetes mellitus with end organ damage	Carbamazepine	Liver diseases without sequelae	Anxiety disorders
**60**	Liver diseases without sequelae	Neurocognitive disorders (Except vascular dementia)	Vascular dementia	Selective serotonin reuptake inhibitor
**61**	Peptic ulcer diseases	Red cell distribution width-coefficient of variation (%)	Diabetes mellitus without end organ damage	Bipolar and related disorders
**62**	Connective tissue diseases	Sexual dysfunctions	Obsessive-compulsive and related disorders	Diabetes mellitus without end organ damage
**63**	Chronic pulmonary diseases	Intraabdominal bleeding	Neurodevelopmental disorders	Trauma- and stressor-related disorders
**64**	Vascular dementia	Metastatic solid tumor	Sleep-wake disorders	Chronic pulmonary diseases
**65**	Cerebrovascular diseases	Hemoglobin (g/dL)	Agomelatine	Vascular dementia
**66**	Peripheral vascular diseases	Agomelatine	Personality disorders	Metformin

**67**	Congestive heart failure	The benzamides	Chronic pulmonary diseases	Liver diseases without sequelae
**68**	Myocardial infarct	Potassium (mg/dL)	Antacids	Sex
**69**	Paraphilic disorders	Alanine aminotransferase (U/L)	Neurocognitive disorders (Except vascular dementia)	Intraabdominal bleeding
**70**	Gender dysphoria	Aripiprazole	Ocular bleeding	Hemopericardium or pericardial bleeding
**71**	Personality disorders	Hematoma or compartment syndrome	Feeding and eating disorders	Myocardial infarct
**72**	Neurocognitive disorders (Except vascular dementia)	Red cell distribution width-standard deviation(fL)	Carbamazepine	Any cancer
**73**	Sexual dysfunctions	Acarbose	Mirtazapine	Congestive heart failure
**74**	Sleep-wake disorders	Thiazolidinediones	Metformin	Peripheral vascular diseases
**75**	Somatic symptom and related disorders	Vascular dementia	Trazodone	Acquired immune deficiency syndrome
**76**	Depressive disorders	Diabetes mellitus with end organ damage	Statins	Cerebrovascular diseases
**77**	Bipolar and related disorders	Fibrates	Sulfonylureas	Hemiplegia
**78**	Schizophrenia spectrum and other psychotic disorders	Diastolic blood pressure (mmHg)	Dipeptidyl peptidase 4 inhibitors	Intracranial bleeding
**79**	Neurodevelopmental disorders	Peptic ulcer diseases	Other bleeding	Diabetes mellitus with end organ damage
**80**	Antacids	Serotonin norepinephrine reuptake inhibitor	Any cancer	Connective tissue diseases
**81**	Amiodarone	Bupropion	Hematoma or compartment syndrome	Ocular bleeding
**82**	Aspirin	Mirtazapine	Liver diseases with sequelae	Hematoma or compartment syndrome
**83**	Diuretics	Disruptive, impulse-control, and conduct disorders	Bupropion	Paraphilic disorders
**84**	Meglitinides	Paraphilic disorders	Fibrates	Peptic ulcer diseases
**85**	Thiazolidinediones	Gender dysphoria	The benzamides	Liver diseases with sequelae
**86**	Acarbose	Platelet (count/uL)	Cerebrovascular diseases	Metastatic solid tumor
**87**	Sulfonylureas	Height (m)	Somatic symptom and related disorders	Thiazolidinediones
**88**	Metformin	Aspirin	Elimination disorders	Gender dysphoria
**89**	Insulins	Red blood cell (count/uL)	Diabetes mellitus with end organ damage	Acarbose
**90**	Statins	Somatic symptom and related disorders	Insulins	Lamotrigine
**91**	Non-benzodiazepines	Antacids	Acarbose	Trazodone
**92**	Benzodiazepines	Calcium channel blockers	Aspirin	Agomelatine
**93**	The benzamides	Dipeptidyl peptidase 4 inhibitors	Acquired immune deficiency syndrome	The benzamides
**94**	Typical antipsychotics	Typical antipsychotics	Intracranial bleeding	Acetylcholinesterase inhibitors
**95**	Bupropion	White blood cell-segment (%)	Diuretics	Statins
**96**	Mirtazapine	White blood cell-eosinophil (%)	Hemiplegia	Fibrates
**97**	Lamotrigine	Statins	Sodium-glucose cotransporter 2 inhibitors	Insulins
**98**	Carbamazepine	Angiotensin converting enzyme inhibitors	Myocardial infarct	Sodium-glucose cotransporter 2 inhibitors
**99**	Aspartate aminotransferase (U/L)	Connective tissue diseases	Peripheral vascular diseases	Dipeptidyl peptidase 4 inhibitors
**100**	Blood urea nitrogen (mg/dL)	Myocardial infarct	Thiazolidinediones	Sulfonylureas
**101**	White blood cell-basophil (%)	Sodium-glucose cotransporter 2 inhibitors	Acetylcholinesterase inhibitors	Meglitinides

**102**	White blood cell-monocyte (%)	Amiodarone	Sexual dysfunctions	Dissociative disorders
**103**	White blood cell-lymphocyte (%)	Other antiplatelet	Connective tissue diseases	Angiotensin converting enzyme inhibitors
**104**	White blood cell-segment (%)	Other bleeding	Congestive heart failure	Diuretics
**105**	Red cell distribution width-coefficient of variation (%)	Meglitinides	Other antiplatelet	Aspirin
**106**	Platelet (count/uL)	Mean corpuscular volume (fL)	Peptic ulcer diseases	Other antiplatelet
**107**	Red cell distribution width-standard deviation(fL)	Sulfonylureas	Meglitinides	Amiodarone
**108**	Mean corpuscular hemoglobin (pg)	Trazodone	Metastatic solid tumor	Obsessive-compulsive and related disorders
**109**	Mean corpuscular volume (fL)	Aspartate aminotransferase (U/L)	Paraphilic disorders	Somatic symptom and related disorders
**110**	Hemoglobin (g/dL)	Peripheral vascular diseases	Gender dysphoria	Elimination disorders
**111**	Red blood cell (count/uL)	Acetylcholinesterase inhibitors	Dissociative disorders	Sexual dysfunctions
**112**	Diastolic blood pressure (mmHg)	Insulins	Intraabdominal bleeding	Neurocognitive disorders (Except vascular dementia)
**113**	Dosing frequency	Natrium (mg/dL)	Hemopericardium or pericardial bleeding	Personality disorders
**114**	Sex	Liver diseases without sequelae	Amiodarone	Other bleeding

Appendix 11 (Supplementary Figure S5). LASSO method for selecting the top 10 features in linear regression, continuous outcomes

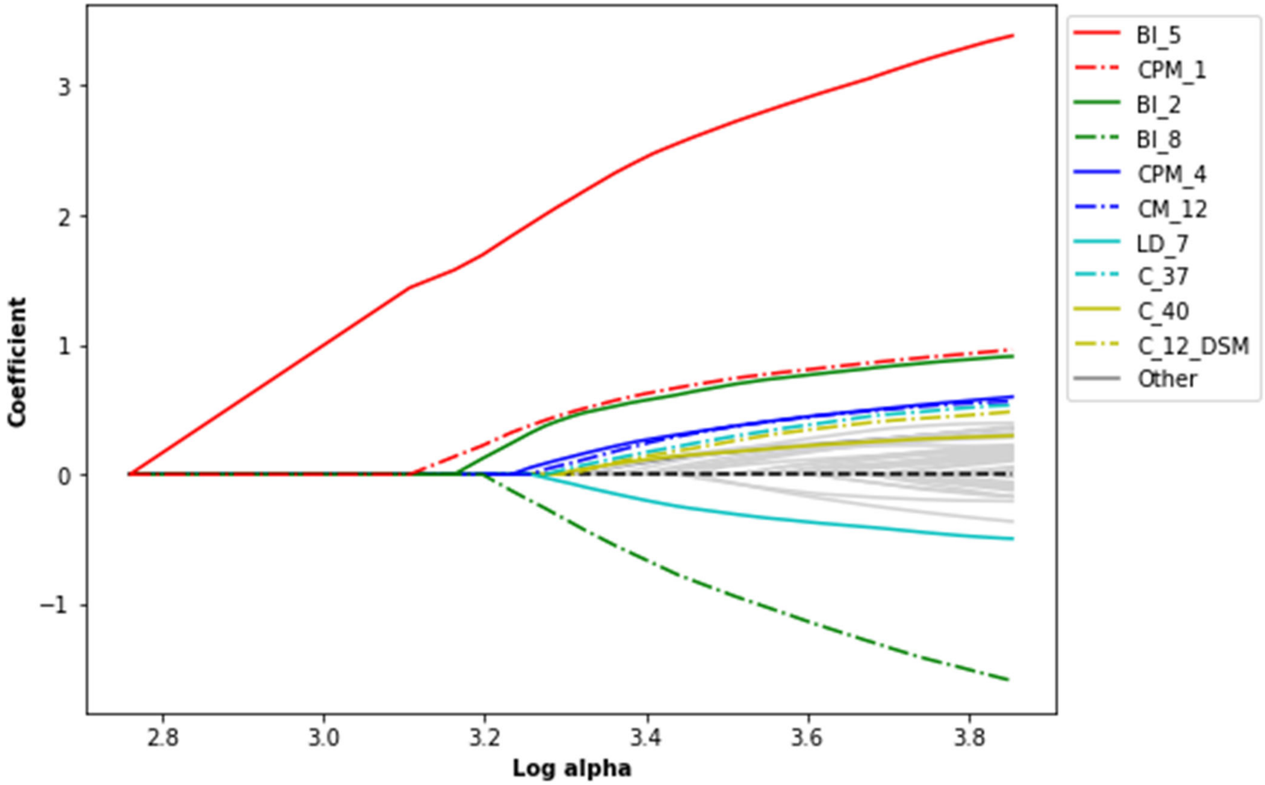

BI_5 = Daily dose, CPM_1 = Valproic acid, BI_2 = Age, BI_8 = Weight (Kg), CPM_4 = Topiramate, CM_12 = Angiotensin receptor blockers, LD_7 = Mean corpuscular hemoglobin concentration, C_37 = Hypertension, C_40 = Ocular bleeding, C_12_DSM = Substance-related and addictive disorders.

Appendix 12 (Supplementary Figure S6). Backward stepwise method for selecting the top 10 features in support vector machine, continuous outcomes

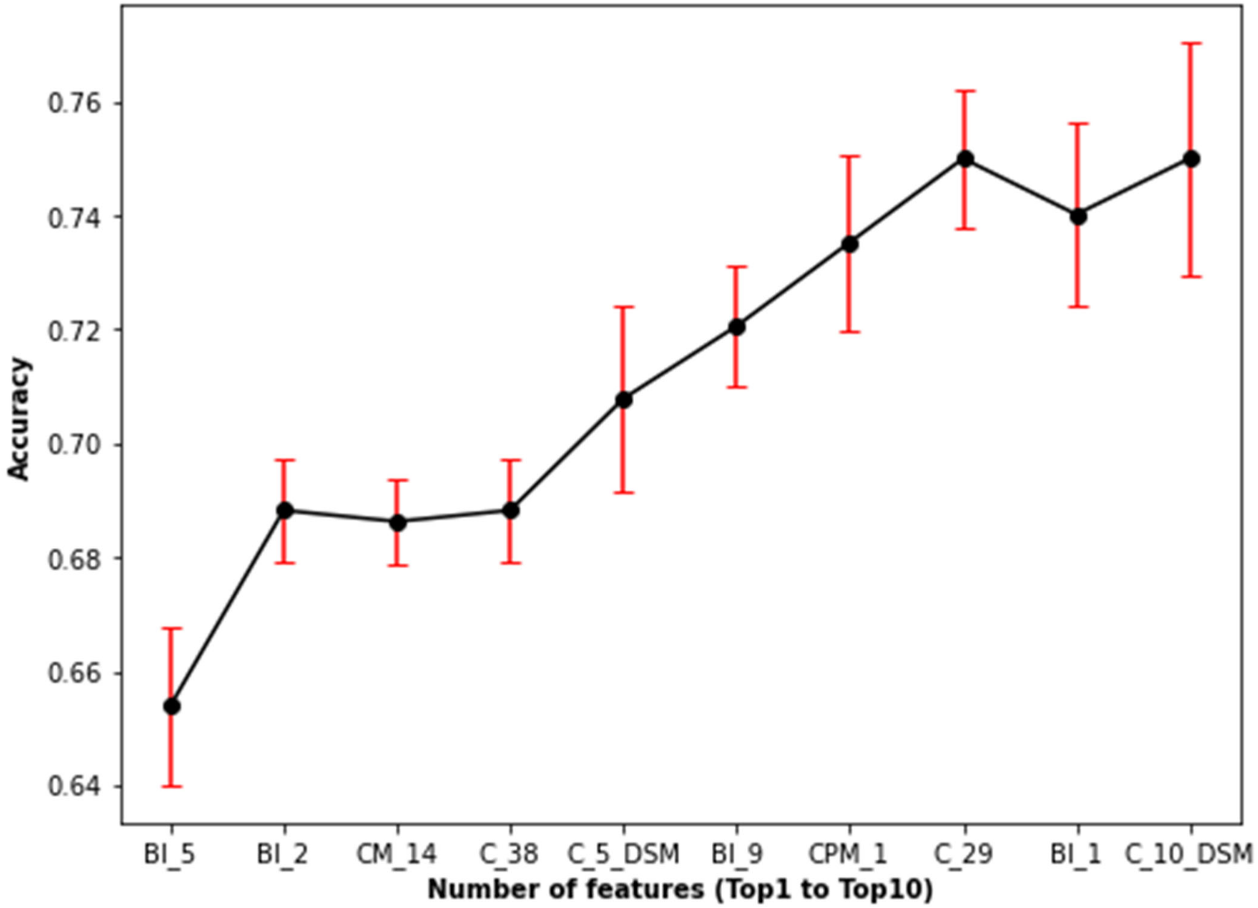

BI_5 = Daily dose, BI_2 = Age, CM_14 = Beta blockers, C_38 = Hyperlipidemia, C_5_DSM = Depressive disorders, BI_9 = Systolic blood pressure, CPM_1 = Valproic acid, C_29 = Diabetes mellitus without end organ damage, BI_1 = Sex, C_10_DSM = Sleep-wake disorders.

Appendix 13 (Supplementary Figure S7). Shapley additive explanations method for selecting the top 10 features in random forests, continuous outcomes

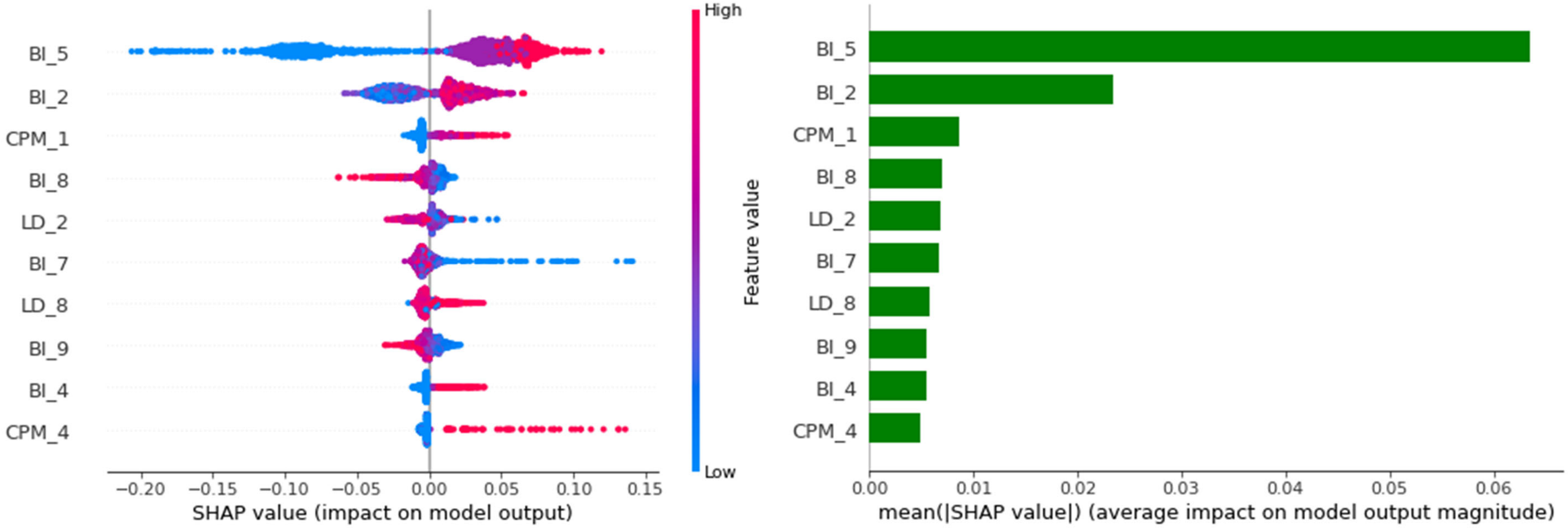

BI_5 = Daily dose, BI_2 = Age, CPM_1 = Valproic acid, BI_8 = Weight, LD_2 = Red blood cell, BI_7 = Height, LD_8 = Red cell distribution width-standard deviation, BI_9 = Systolic blood pressure, BI_4 = Last dose, CPM_4 = Topiramate.

Appendix 14 (Supplementary Figure S8). Shapley additive explanations method for selecting the top 10 features in extreme gradient boost, continuous outcomes

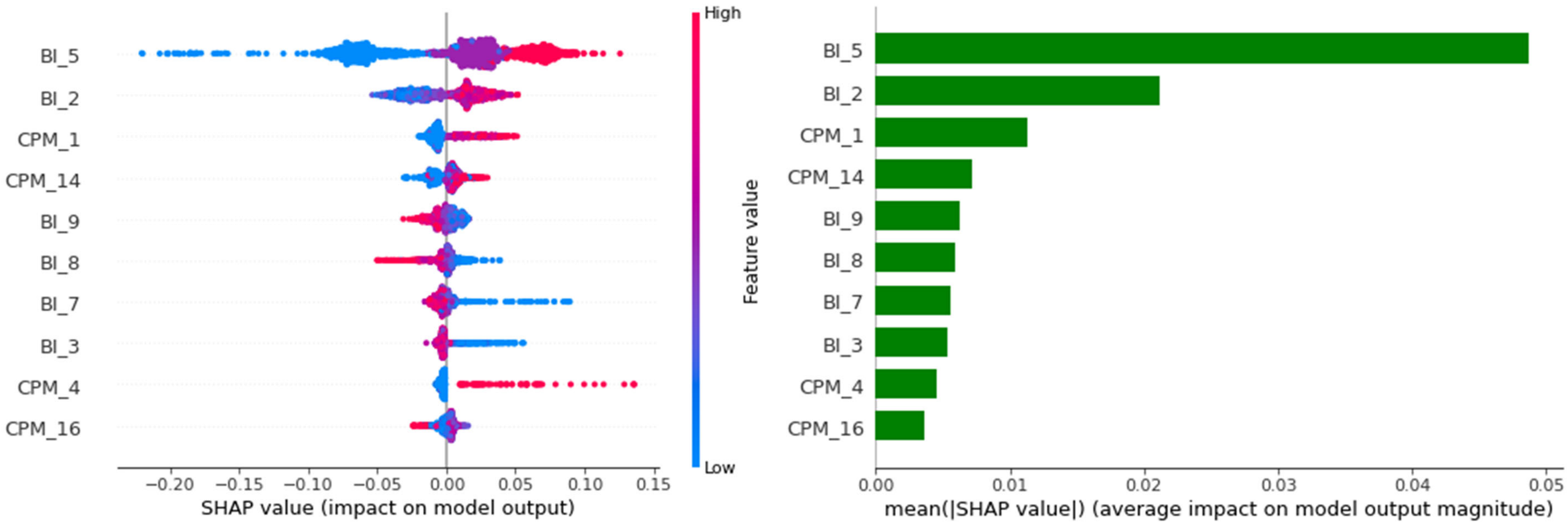

BI_5 = Daily dose, BI_2 = Age, CPM_1 = Valproic acid, CPM_14 = The -pines, BI_9 = Systolic blood pressure, BI_8 = Weight, BI_7 = Height, BI_3 = Time interval, CPM_4 = Topiramate, CPM_16 = Benzodiazepines.

Appendix 15 (Supplementary Table S7). 114 feature importance ranking results of the 4 different machine learning algorithms in continuous outcomes

Rank	Linear regression	Support vector machine	Random forests	Extreme gradient boosting
1	Daily dose	Daily dose	Daily dose	Daily dose
2	Valproic acid	Age	Age	Age
3	Age	Beta blockers	Valproic acid	Valproic acid
4	Weight (Kg)	Hyperlipidemia	Weight (Kg)	The -pines
5	Topiramate	Depressive disorders	Red blood cell (count/uL)	Systolic blood pressure (mmHg)
6	Angiotensin receptor blockers	Systolic blood pressure (mmHg)	Height (m)	Weight (Kg)
7	Mean corpuscular hemoglobin concentration (gHb/dL)	Valproic acid	Red cell distribution width-standard deviation(fL)	Height (m)
8	Hypertension	Diabetes mellitus without end organ damage	Systolic blood pressure (mmHg)	Time interval
9	Ocular bleeding	Sex	Last dose	Topiramate
10	Substance-related and addictive disorders	Sleep-wake disorders	Topiramate	Benzodiazepines
11	Acetylcholinesterase inhibitors	Dosing frequency	The -pines	Red cell distribution width-standard deviation(fL)
12	Depressive disorders	The -pines	Time interval	Red blood cell (count/uL)
13	The -pines	Substance-related and addictive disorders	Hypertension	Non-steroidal anti-inflammatory drugs
14	Neurocognitive disorders (Except vascular dementia)	Diabetes mellitus with end organ damage	Platelet (count/uL)	Platelet (count/uL)
15	Trauma- and stressor-related disorders	Obsessive-compulsive and related disorders	Benzodiazepines	Substance-related and addictive disorders
16	Renal diseases	Liver diseases with sequelae	Non-steroidal anti-inflammatory drugs	The -dones
17	Fibrates	Gastrointestinal bleeding	Substance-related and addictive disorders	Beta blockers
18	Last dose	Chronic pulmonary diseases	Red cell distribution width-coefficient of variation (%)	Serum creatinine (mg/dL)
19	Beta blockers	Vascular dementia	Mean corpuscular hemoglobin concentration (gHb/dL)	Last dose
20	Systolic blood pressure (mmHg)	White blood cell-eosinophil (%)	White blood cell (count/uL)	Hypertension
21	Potassium (mg/dL)	Non-benzodiazepines	The -dones	Mean corpuscular hemoglobin concentration (gHb/dL)
22	Acquired immune deficiency syndrome	The benzamides	Serum creatinine (mg/dL)	Red cell distribution width-coefficient of variation (%)
23	Agomelatine	Neurocognitive disorders (Except vascular dementia)	Hemoglobin (g/dL)	Dosing frequency
24	Metastatic solid tumor	Dipeptidyl peptidase 4 inhibitors	Beta blockers	Depressive disorders
25	Natrium (mg/dL)	Last dose	Natrium (mg/dL)	White blood cell-lymphocyte (%)
26	Trazodone	Potassium (mg/dL)	Aspartate aminotransferase (U/L)	Aspartate aminotransferase (U/L)
27	Obsessive-compulsive and related disorders	Hemoglobin (g/dL)	Mean corpuscular volume (fL)	Natrium (mg/dL)
28	Non-steroidal anti-inflammatory drugs	Neurodevelopmental disorders	Diastolic blood pressure (mmHg)	Hemoglobin (g/dL)
29	Anxiety disorders	Selective serotonin reuptake inhibitor	Blood urea nitrogen (mg/dL)	White blood cell (count/uL)
30	The benzamides	Blood urea nitrogen (mg/dL)	Hematocrit (%)	Mean corpuscular hemoglobin (pg)
31	Sulfonylureas	Diastolic blood pressure (mmHg)	Hyperlipidemia	Hyperlipidemia
32	Elimination disorders	Cerebrovascular diseases	Potassium (mg/dL)	Angiotensin receptor blockers

**33**	White blood cell-basophil (%)	Acquired immune deficiency syndrome	Alanine aminotransferase (U/L)	Alanine aminotransferase (U/L)
**34**	Other bleeding	Non-steroidal anti-inflammatory drugs	Mean corpuscular hemoglobin (pg)	Potassium (mg/dL)
**35**	Red cell distribution width-standard deviation(fL)	Liver diseases without sequelae	White blood cell-monocyte (%)	White blood cell-segment (%)
**36**	Serum creatinine (mg/dL)	Aripiprazole	White blood cell-eosinophil (%)	White blood cell-monocyte (%)
**37**	Somatic symptom and related disorders	White blood cell-basophil (%)	White blood cell-lymphocyte (%)	Blood urea nitrogen (mg/dL)
**38**	Time interval	Somatic symptom and related disorders	Anxiety disorders	Diastolic blood pressure (mmHg)
**39**	Dipeptidyl peptidase 4 inhibitors	Trauma- and stressor-related disorders	White blood cell-segment (%)	White blood cell-eosinophil (%)
**40**	Aripiprazole	Serum creatinine (mg/dL)	Angiotensin receptor blockers	Sex
**41**	Diabetes mellitus without end organ damage	Other bleeding	Depressive disorders	Non-benzodiazepines
**42**	Diuretics	The -dones	Liver diseases without sequelae	Diabetes mellitus without end organ damage
**43**	The -dones	Statins	White blood cell-basophil (%)	Hematocrit (%)
**44**	Acarbose	Height (m)	Trauma- and stressor-related disorders	Sleep-wake disorders
**45**	Any cancer	Red cell distribution width-standard deviation(fL)	Typical antipsychotics	Vascular dementia
**46**	White blood cell-eosinophil (%)	White blood cell (count/uL)	Diabetes mellitus without end organ damage	Antacids
**47**	Neurodevelopmental disorders	Platelet (count/uL)	Dosing frequency	Liver diseases without sequelae
**48**	Statins	Dissociative disorders	Selective serotonin reuptake inhibitor	Feeding and eating disorders
**49**	Angiotensin converting enzyme inhibitors	Hematocrit (%)	Mirtazapine	Neurocognitive disorders (Except vascular dementia)
**50**	Other antiplatelet	Hemopericardium or pericardial bleeding	Gastrointestinal bleeding	Selective serotonin reuptake inhibitor
**51**	Height (m)	Intraabdominal bleeding	Neurocognitive disorders (Except vascular dementia)	Carbamazepine
**52**	Gastrointestinal bleeding	Congestive heart failure	Non-benzodiazepines	Aripiprazole
**53**	Hematoma or compartment syndrome	Mean corpuscular hemoglobin (pg)	Neurodevelopmental disorders	White blood cell-basophil (%)
**54**	Hemopericardium or pericardial bleeding	Calcium channel blockers	Aripiprazole	Ocular bleeding
**55**	Intraabdominal bleeding	Antacids	Schizophrenia spectrum and other psychotic disorders	Mean corpuscular volume (fL)
**56**	Intracranial bleeding	Peptic ulcer diseases	Bipolar and related disorders	Anxiety disorders
**57**	Hyperlipidemia	Hemiplegia	Sex	Typical antipsychotics
**58**	Liver diseases with sequelae	Angiotensin converting enzyme inhibitors	Vascular dementia	Neurodevelopmental disorders
**59**	Hemiplegia	Sulfonylureas	Ocular bleeding	Personality disorders
**60**	Diabetes mellitus with end organ damage	Aspirin	Obsessive-compulsive and related disorders	Trauma- and stressor-related disorders
**61**	Liver diseases without sequelae	Paraphilic disorders	Antacids	Aspirin
**62**	Peptic ulcer diseases	Metformin	Agomelatine	Bipolar and related disorders
**63**	Connective tissue diseases	Renal diseases	Feeding and eating disorders	Calcium channel blockers
**64**	Chronic pulmonary diseases	Disruptive, impulse-control, and conduct disorders	Diuretics	Cerebrovascular diseases

**65**	Vascular dementia	Ocular bleeding	Sleep-wake disorders	Schizophrenia spectrum and other psychotic disorders
**66**	Cerebrovascular diseases	Mean corpuscular volume (fL)	Serotonin norepinephrine reuptake inhibitor	Diuretics
**67**	Peripheral vascular diseases	Elimination disorders	Calcium channel blockers	Renal diseases
**68**	Congestive heart failure	Red cell distribution width-coefficient of variation (%)	Carbamazepine	Acetylcholinesterase inhibitors
**69**	Myocardial infarct	Hematoma or compartment syndrome	Personality disorders	Liver diseases with sequelae
**70**	Paraphilic disorders	Feeding and eating disorders	Trazodone	Angiotensin converting enzyme inhibitors
**71**	Gender dysphoria	Connective tissue diseases	Disruptive, impulse-control, and conduct disorders	Sulfonylureas
**72**	Feeding and eating disorders	Agomelatine	Elimination disorders	Obsessive-compulsive and related disorders
**73**	Dissociative disorders	White blood cell-lymphocyte (%)	Aspirin	Any cancer
**74**	Personality disorders	Gender dysphoria	Fibrates	Chronic pulmonary diseases
**75**	Sexual dysfunctions	Metastatic solid tumor	Chronic pulmonary diseases	Elimination disorders
**76**	Sleep-wake disorders	Intracranial bleeding	Lamotrigine	The benzamides
**77**	Bipolar and related disorders	Acarbose	Myocardial infarct	Agomelatine
**78**	Schizophrenia spectrum and other psychotic disorders	Meglitinides	Insulins	Diabetes mellitus with end organ damage
**79**	Disruptive, impulse-control, and conduct disorders	Typical antipsychotics	Metformin	Lamotrigine
**80**	Antacids	Sodium-glucose cotransporter 2 inhibitors	Renal diseases	Sexual dysfunctions
**81**	Amiodarone	Myocardial infarct	Angiotensin converting enzyme inhibitors	Hematoma or compartment syndrome
**82**	Aspirin	Amiodarone	Sulfonylureas	Intracranial bleeding
**83**	Calcium channel blockers	Bupropion	Cerebrovascular diseases	Hemiplegia
**84**	Meglitinides	Serotonin norepinephrine reuptake inhibitor	The benzamides	Acquired immune deficiency syndrome
**85**	Thiazolidinediones	Carbamazepine	Other bleeding	Hemopericardium or pericardial bleeding
**86**	Sodium-glucose cotransporter 2 inhibitors	Hypertension	Statins	Gastrointestinal bleeding
**87**	Metformin	Benzodiazepines	Acetylcholinesterase inhibitors	Metastatic solid tumor
**88**	Insulins	Aspartate aminotransferase (U/L)	Acarbose	Peptic ulcer diseases
**89**	Non-benzodiazepines	Diuretics	Liver diseases with sequelae	Connective tissue diseases
**90**	Benzodiazepines	Thiazolidinediones	Any cancer	Intraabdominal bleeding
**91**	Typical antipsychotics	Sexual dysfunctions	Bupropion	Thiazolidinediones
**92**	Bupropion	Red blood cell (count/uL)	Acquired immune deficiency syndrome	Peripheral vascular diseases
**93**	Mirtazapine	Lamotrigine	Diabetes mellitus with end organ damage	Dipeptidyl peptidase 4 inhibitors
**94**	Serotonin norepinephrine reuptake inhibitor	Any cancer	Metastatic solid tumor	Serotonin norepinephrine reuptake inhibitor
**95**	Selective serotonin reuptake inhibitor	Angiotensin receptor blockers	Other antiplatelet	Trazodone
**96**	Lamotrigine	Insulins	Somatic symptom and related disorders	Mirtazapine
**97**	Carbamazepine	White blood cell-segment (%)	Dipeptidyl peptidase 4 inhibitors	Bupropion
**98**	Alanine aminotransferase (U/L)	Fibrates	Intracranial bleeding	Statins

**99**	Aspartate aminotransferase (U/L)	Alanine aminotransferase (U/L)	Hematoma or compartment syndrome	Fibrates
**100**	Blood urea nitrogen (mg/dL)	Natrium (mg/dL)	Dissociative disorders	Insulins
**101**	White blood cell-monocyte (%)	Other antiplatelet	Hemiplegia	Metformin
**102**	White blood cell-lymphocyte (%)	Peripheral vascular diseases	Connective tissue diseases	Sodium-glucose cotransporter 2 inhibitors
**103**	White blood cell-segment (%)	Mean corpuscular hemoglobin concentration (gHb/dL)	Gender dysphoria	Acarbose
**104**	Red cell distribution width-coefficient of variation (%)	Mirtazapine	Meglitinides	Congestive heart failure
**105**	Platelet (count/uL)	Topiramate	Peptic ulcer diseases	Meglitinides
**106**	Mean corpuscular hemoglobin (pg)	Trazodone	Sodium-glucose cotransporter 2 inhibitors	Other antiplatelet
**107**	Mean corpuscular volume (fL)	White blood cell-monocyte (%)	Amiodarone	Amiodarone
**108**	Hematocrit (%)	Personality disorders	Peripheral vascular diseases	Disruptive, impulse-control, and conduct disorders
**109**	Hemoglobin (g/dL)	Acetylcholinesterase inhibitors	Congestive heart failure	Somatic symptom and related disorders
**110**	Red blood cell (count/uL)	Bipolar and related disorders	Intraabdominal bleeding	Dissociative disorders
**111**	White blood cell (count/uL)	Time interval	Hemopericardium or pericardial bleeding	Gender dysphoria
**112**	Diastolic blood pressure (mmHg)	Anxiety disorders	Sexual dysfunctions	Paraphilic disorders
**113**	Dosing frequency	Weight (Kg)	Paraphilic disorders	Myocardial infarct
**114**	Sex	Schizophrenia spectrum and other psychotic disorders	Thiazolidinediones	Other bleeding

Appendix 16 (Supplementary Table S8). Detail information of binary outcomes of logistic regression algorithm under different feature combinations

**Model (LogR)**	**TP**	**TN**	**FP**	**FN**	**TPR (Sensitivity)**	**TNR (Specificity)**	**PPV (Precision)**	**NPV**	**AUC-ROC**	**F-score**	**Accuracy**
**Model P**	118 (112-124)	31 (25-36)	40 (35-46)	15 (9-21)	0.886 (0.839-0.932)	0.431 (0.356-0.506)	0.745 (0.726-0.764)	0.675 (0.600-0.750)	0.748 (0.732-0.764)	0.809 (0.792-0.826)	0.727 (0.707-0.748)
**Model 1**	125 (124-127)	21 (18-23)	50 (48-53)	8 (6-9)	0.941 (0.929-0.954)	0.290 (0.252-0.328)	0.713 (0.701-0.725)	0.725 (0.677-0.774)	0.753 (0.741-0.766)	0.811 (0.801-0.822)	0.715 (0.698-0.731)
**Model 2**	116 (113-119)	28 (24-32)	43 (39-47)	17 (14-20)	0.874 (0.852-0.895)	0.400 (0.343-0.457)	0.732 (0.715-0.749)	0.628 (0.588-0.669)	0.734 (0.721-0.747)	0.796 (0.785-0.808)	0.709 (0.691-0.726)
**Model 3**	121 (120-123)	27 (26-28)	44 (43-45)	12 (10-13)	0.913 (0.899-0.927)	0.380 (0.363-0.398)	0.734 (0.726-0.742)	0.700 (0.659-0.741)	0.761 (0.751-0.771)	0.814 (0.804-0.824)	0.727 (0.713-0.741)

Abbreviations: AUC-ROC, area under the curve of receiver operator characteristic; FN, false negative; FP, false positive; LogR, Logistic regression; NPV, negative predictive value; PPV, positive predictive value; TN, true negative; TP, true positive; TNR, true negative rate; TPR, true positive rate.

Model P = primary model using all features; Model 1 = model using basic information as features; Model 2 = model using basic information and concomitant psychotropic medications as features; Model 3 = model using the top 10 features of different algorithms as features.

Appendix 17 (Supplementary Table S9). Detail information of binary outcomes of support vector machine algorithm under different feature combinations

Model (SVM)	TP	TN	FP	FN	TPR (Sensitivity)	TNR (Specificity)	PPV (Precision)	NPV	AUC-ROC	F-score	Accuracy
**Model P**	125 (121-129)	23 (17-29)	48 (42-54)	8 (4-12)	0.940 (0.911-0.969)	0.324 (0.241-0.407)	0.723 (0.702-0.744)	0.747 (0.675-0.819)	0.759 (0.744-0.774)	0.817 (0.806-0.828)	0.725 (0.706-0.745)
**Model 1**	123 (120-126)	21 (17-24)	50 (47-54)	10 (7-13)	0.925 (0.901-0.949)	0.290 (0.238-0.342)	0.709 (0.690-0.728)	0.673 (0.576-0.770)	0.766 (0.747-0.786)	0.803 (0.783-0.823)	0.704 (0.673-0.735)
**Model 2**	120 (119-122)	26 (21-32)	45 (39-50)	13 (11-14)	0.904 (0.894-0.914)	0.369 (0.293-0.445)	0.729 (0.706-0.752)	0.669 (0.625-0.714)	0.775 (0.754-0.797)	0.807 (0.793-0.821)	0.718 (0.693-0.743)
**Model 3**	121 (119-122)	28 (23-32)	43 (39-48)	12 (11-14)	0.908 (0.896-0.920)	0.392 (0.327-0.456)	0.737 (0.718-0.756)	0.694 (0.671-0.717)	0.771 (0.762-0.779)	0.814 (0.805-0.823)	0.728 (0.711-0.746)

Abbreviations: AUC-ROC, area under the curve of receiver operator characteristic; FN, false negative; FP, false positive; NPV, negative predictive value; PPV, positive predictive value; SVM, support vector machine; TN, true negative; TP, true positive; TNR, true negative rate; TPR, true positive rate.
Model P = primary model using all features; Model 1 = model using basic information as features; Model 2 = model using basic information and concomitant psychotropic medications as features; Model 3 = model using the top 10 features of different algorithms as features.

Appendix 18 (Supplementary Table S10). Detail information of binary outcomes of random forests algorithm under different feature combinations

Model (RF)	TP	TN	FP	FN	TPR (Sensitivity)	TNR (Specificity)	PPV (Precision)	NPV	AUC-ROC	F-score	Accuracy
**Model P**	127 (126-129)	16 (10-22)	55 (49-61)	6 (4-7)	0.958 (0.947-0.969)	0.220 (0.134-0.305)	0.697 (0.676-0.719)	0.728 (0.659-0.797)	0.784 (0.754-0.814)	0.807 (0.794-0.820)	0.701 (0.675-0.727)
**Model 1**	122 (120-125)	24 (20-28)	47 (43-51)	11 (8-13)	0.920 (0.901-0.940)	0.341 (0.286-0.395)	0.723 (0.703-0.744)	0.694 (0.613-0.775)	0.767 (0.759-0.775)	0.810 (0.790-0.830)	0.719 (0.688-0.749)
**Model 2**	125 (124-127)	21 (17-25)	50 (46-54)	8 (6-9)	0.941 (0.929-0.954)	0.299 (0.244-0.353)	0.716 (0.701-0.731)	0.731 (0.688-0.773)	0.796 (0.786-0.806)	0.813 (0.803-0.823)	0.718 (0.700-0.735)
**Model 3**	119 (116-123)	26 (21-31)	45 (40-50)	14 (10-17)	0.896 (0.871-0.921)	0.369 (0.298-0.440)	0.727 (0.706-0.749)	0.655 (0.598-0.712)	0.785 (0.777-0.792)	0.803 (0.786-0.819)	0.713 (0.687-0.739)

Abbreviations: AUC-ROC, area under the curve of receiver operator characteristic; FN, false negative; FP, false positive; NPV, negative predictive value; PPV, positive predictive value; RF, random forests; TN, true negative; TP, true positive; TNR, true negative rate; TPR, true positive rate.

Model P = primary model using all features; Model 1 = model using basic information as features; Model 2 = model using basic information and concomitant psychotropic medications as features; Model 3 = model using the top 10 features of different algorithms as features.

Appendix 19 (Supplementary Table S11). Detail information of binary outcomes of extreme gradient boosting algorithm under different feature combinations

**Model (XGBoost)**	**TP**	**TN**	**FP**	**FN**	**TPR (Sensitivity)**	**TNR (Specificity)**	**PPV (Precision)**	**NPV**	**AUC-ROC**	**F-score**	**Accuracy**
**Model P**	120 (116-125)	27 (24-29)	44 (42-47)	13 (8-17)	0.904 (0.870-0.938)	0.377 (0.344-0.411)	0.731 (0.721-0.741)	0.681 (0.607-0.756)	0.775 (0.741-0.810)	0.808 (0.792-0.825)	0.721 (0.700-0.741)
**Model 1**	120 (118-121)	25 (22-28)	46 (43-49)	13 (12-15)	0.899 (0.887-0.912)	0.346 (0.304-0.389)	0.721 (0.706-0.735)	0.647 (0.601-0.693)	0.756 (0.732-0.780)	0.800 (0.788-0.812)	0.707 (0.688-0.726)
**Model 2**	122 (118-126)	27 (23-31)	44 (40-48)	11 (7-15)	0.916 (0.887-0.944)	0.383 (0.326-0.440)	0.736 (0.716-0.755)	0.710 (0.629-0.791)	0.778 (0.750-0.806)	0.816 (0.797-0.835)	0.730 (0.702-0.759)
**Model 3**	116 (113-120)	31 (28-34)	40 (37-43)	17 (13-20)	0.875 (0.852-0.899)	0.434 (0.389-0.479)	0.743 (0.731-0.756)	0.651 (0.616-0.685)	0.766 (0.748-0.785)	0.804 (0.793-0.815)	0.722 (0.707-0.736)

Abbreviations: AUC-ROC, area under the curve of receiver operator characteristic; FN, false negative; FP, false positive; NPV, negative predictive value; PPV, positive predictive value; TN, true negative; TP, true positive; TNR, true negative rate; TPR, true positive rate; XGBoost, extreme gradient boosting. Model P = primary model using all features; Model 1 = model using basic information as features; Model 2 = model using basic information and concomitant psychotropic medications as features; Model 3 = model using the top 10 features of different algorithms as features.

Appendix 20 (Supplementary Table S12). Detail information of continuous outcomes of linear regression algorithm under different feature combinations

Model (LinR)	MAE	MSE	RMSE	R^2	Accuracy
**Model P**	0.159 (0.156-0.162)	0.038 (0.037-0.040)	0.196 (0.193-0.199)	0.209 (0.187-0.231)	0.690 (0.677-0.703)
**Model 1**	0.159 (0.158-0.160)	0.039 (0.039-0.039)	0.197 (0.196-0.198)	0.199 (0.192-0.207)	0.670 (0.654-0.685)
**Model 2**	0.157 (0.155-0.159)	0.039 (0.038-0.039)	0.196 (0.195-0.198)	0.207 (0.197-0.217)	0.670 (0.658-0.681)
**Model 3**	0.157 (0.155-0.159)	0.038 (0.038-0.039)	0.196 (0.194-0.197)	0.211 (0.200-0.223)	0.691 (0.676-0.706)

Abbreviations: LinR, linear regression; MAE, mean absolute error; MSE, mean-square error; RMSE, root-mean-square error.

Model P = primary model using all features; Model 1 = model using basic information as features; Model 2 = model using basic information and concomitant psychotropic medications as features; Model 3 = model using the top 10 features of different algorithms as features.

Appendix 21 (Supplementary Table S13). Detail information of continuous outcomes of support vector machine algorithm under different feature combinations

Model (SVM)	MAE	MSE	RMSE	R^2	Accuracy
**Model P**	0.140 (0.134-0.145)	0.031 (0.028-0.033)	0.175 (0.167-0.183)	0.370 (0.312-0.427)	0.751 (0.710-0.792)
**Model 1**	0.150 (0.147-0.152)	0.036 (0.034-0.037)	0.189 (0.186-0.192)	0.266 (0.241-0.291)	0.716 (0.705-0.726)
**Model 2**	0.145 (0.139-0.150)	0.032 (0.031-0.034)	0.180 (0.175-0.185)	0.333 (0.294-0.372)	0.735 (0.713-0.758)
**Model 3**	0.160 (0.158-0.161)	0.040 (0.038-0.041)	0.200 (0.196-0.203)	0.179 (0.149-0.208)	0.690 (0.674-0.706)

Abbreviations: MAE, mean absolute error; MSE, mean-square error; RMSE, root-mean-square error; SVM, support vector machine.
Model P = primary model using all features; Model 1 = model using basic information as features; Model 2 = model using basic information and concomitant psychotropic medications as features; Model 3 = model using the top 10 features of different algorithms as features.

Appendix 22 (Supplementary Table S14). Detail information of continuous outcomes of random forests algorithm under different feature combinations

Model (RF)	MAE	MSE	RMSE	R^2	Accuracy
**Model P**	0.152 (0.147-0.156)	0.035 (0.034-0.037)	0.188 (0.184-0.192)	0.273 (0.245-0.301)	0.680 (0.665-0.696)
**Model 1**	0.154 (0.153-0.155)	0.037 (0.036-0.037)	0.191 (0.190-0.193)	0.246 (0.237-0.255)	0.696 (0.681-0.711)
**Model 2**	0.150 (0.146-0.155)	0.035 (0.033-0.036)	0.186 (0.182-0.190)	0.287 (0.259-0.315)	0.681 (0.660-0.703)
**Model 3**	0.155 (0.153-0.158)	0.038 (0.037-0.039)	0.194 (0.192-0.197)	0.222 (0.203-0.242)	0.687 (0.665-0.710)

Abbreviations: MAE, mean absolute error; MSE, mean-square error; RF, random forests; RMSE, root-mean-square error.

Model P = primary model using all features; Model 1 = model using basic information as features; Model 2 = model using basic information and concomitant psychotropic medications as features; Model 3 = model using the top 10 features of different algorithms as features.

Appendix 23 (Supplementary Table S15). Detail information of continuous outcomes of extreme gradient boosting algorithm under different feature combinations

Model (XGBoost)	MAE	MSE	RMSE	R^2	Accuracy
**Model P**	0.155 (0.150-0.159)	0.037 (0.036-0.039)	0.193 (0.189-0.196)	0.236 (0.206-0.266)	0.683 (0.671-0.695)
**Model 1**	0.155 (0.154-0.157)	0.038 (0.037-0.038)	0.195 (0.194-0.196)	0.220 (0.212-0.229)	0.683 (0.664-0.702)
**Model 2**	0.153 (0.149-0.158)	0.037 (0.035-0.038)	0.192 (0.188-0.195)	0.245 (0.215-0.274)	0.689 (0.660-0.718)
**Model 3**	0.159 (0.158-0.161)	0.039 (0.039-0.040)	0.198 (0.197-0.200)	0.191 (0.178-0.204)	0.679 (0.670-0.689)

Abbreviations: MAE, mean absolute error; MSE, mean-square error; RMSE, root-mean-square error; XGBoost, extreme gradient boosting.

Model P = primary model using all features; Model 1 = model using basic information as features; Model 2 = model using basic information and concomitant psychotropic medications as features; Model 3 = model using the top 10 features of different algorithms as features.

Appendix 24 (Supplementary Figure S9). Comparison of accuracy performance between the primary model and different new models under different algorithms.

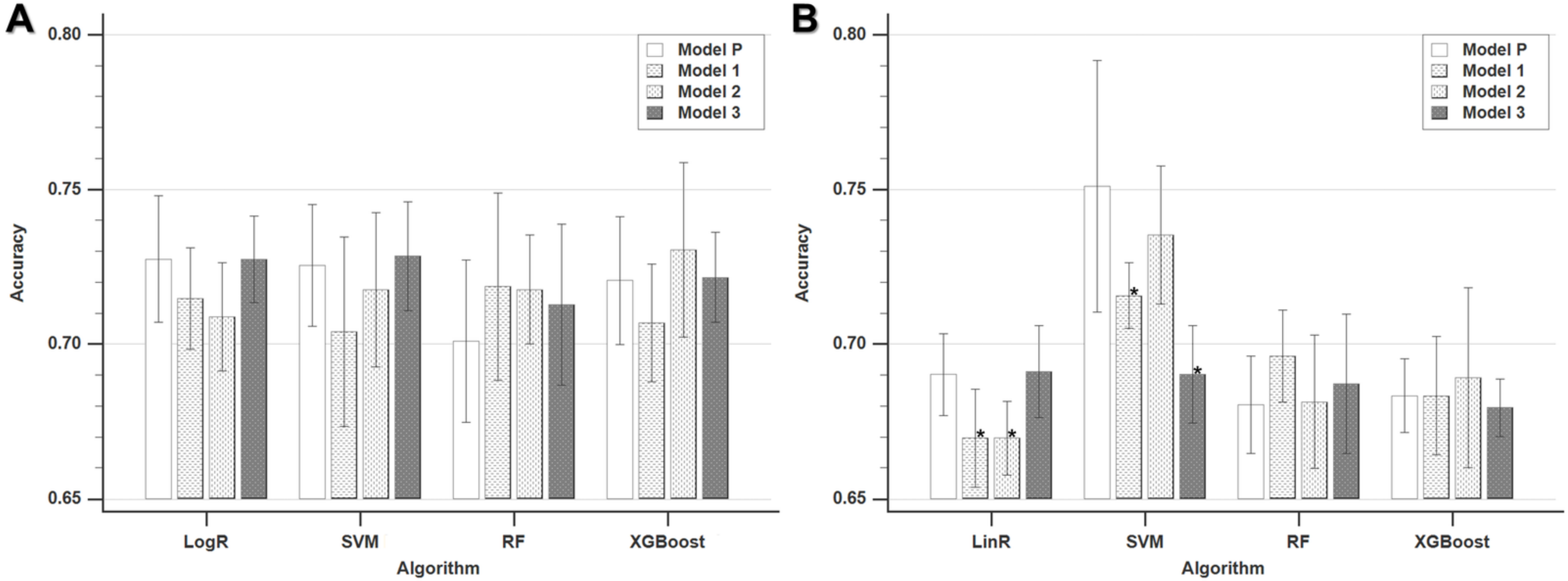

(A) binary prediction, (B) continuous prediction. LinR = linear regression; LogR = logistic regression; Model P = primary model using all features; Model 1 = model using basic in-formation as features; Model 2 = model using basic information and concomitant psychotropic medications as features; Model 3 = model using the top 10 features of different algorithms as features; RF = random forests; SVM = support vector machine; XGBoost = extreme gradient boosting.
